# Colistin and its role in the Era of antibiotic resistance: an extended review (2000–2019)

**DOI:** 10.1080/22221751.2020.1754133

**Published:** 2020-05-06

**Authors:** Mohamed Abd El-Gawad El-Sayed Ahmed, Lan-Lan Zhong, Cong Shen, Yongqiang Yang, Yohei Doi, Guo-Bao Tian

**Affiliations:** a Department of Microbiology, Zhongshan School of Medicine, Sun Yat-sen University, Guangzhou, People’s Republic of China; b Key Laboratory of Tropical Diseases Control, Sun Yat-sen University, Ministry of Education, Guangzhou, People’s Republic of China; c Department of Microbiology and Immunology, Faculty of Pharmaceutical Sciences and Drug Manufacturing, Misr University for Science and Technology (MUST), Cairo, Egypt; d University of Pittsburgh School of Medicine, Pittsburgh, PA, USA; e Department of Microbiology and Infectious Diseases, Fujita Health University, School of Medicine, Aichi, Japan

**Keywords:** Colistin, multidrug resistance, two-component systems, heteroresistance, MCR-1

## Abstract

Increasing antibiotic resistance in multidrug-resistant (MDR) Gram-negative bacteria (MDR-GNB) presents significant health problems worldwide, since the vital available and effective antibiotics, including; broad-spectrum penicillins, fluoroquinolones, aminoglycosides, and β-lactams, such as; carbapenems, monobactam, and cephalosporins; often fail to fight MDR Gram-negative pathogens as well as the absence of new antibiotics that can defeat these “superbugs”. All of these has prompted the reconsideration of old drugs such as polymyxins that were reckoned too toxic for clinical use. Only two polymyxins, polymyxin E (colistin) and polymyxin B, are currently commercially available. Colistin has re-emerged as a last-hope treatment in the mid-1990s against MDR Gram-negative pathogens due to the development of extensively drug-resistant GNB. Unfortunately, rapid global resistance towards colistin has emerged following its resurgence. Different mechanisms of colistin resistance have been characterized, including intrinsic, mutational, and transferable mechanisms.

In this review, we intend to discuss the progress over the last two decades in understanding the alternative colistin mechanisms of action and different strategies used by bacteria to develop resistance against colistin, besides providing an update about what is previously recognized and what is novel concerning colistin resistance.

## Introduction and overview of polymyxins

The escalating increase in antibiotic resistance that launched in the 1970s among Gram-negative bacteria is becoming a critical global crisis [1]. The main issue is that we are running out of possible alternatives that can be used to treat specific pathogens, in particular those that cause hospital-acquired infection, but with the potential to spread throughout the community, indicating that antibiotic resistance could become a global catastrophe that shows no sign of abating [2].

Unfortunately, multidrug-resistant (MDR), extensively drug-resistant (XDR), and pan-drug-resistant (PDR) strains of *Escherichia coli*, *Klebsiella pneumoniae*, *Acinetobacter baumannii*, and *Pseudomonas aeruginosa* are globally found to harbour multiple resistance mechanisms [3,4]. The world is now facing a formidable and growing menace from the emergence of bacteria that are resistant to almost all available antibiotics [2,5,6]. As highlighted by the Infectious Diseases Society of America in the “Bad Bugs, No Drugs” paper, “as antibiotic discovery stagnates, a public health crisis brews” [7].

Regrettably, very little has been accomplished in the pharmaceutical industry to impede this problem. The absence of new antibiotics against these “superbugs” in the near future due to the drying up of the antibiotic discovery pipeline, has led to renewed interest in reviving older antibiotics that were deemed too toxic for clinical use, in particular, the polymyxins (colistin and polymyxin B), to be used as “last resort” antimicrobials [8,9]. In this context, the use of colistin has re-emerged, mainly for use against infections caused by MDR Gram-negative pathogens [1].

Polymyxins, a structurally distinct class of nonribosomal, cyclic oligopeptides antimicrobials, include five chemically distinguished compounds (polymyxins A, B, C, D, and E) of which polymyxin B and colistin (polymyxin E) are the only two polymyxins currently available on the market [1,10,11]. In 1947 in Japan, Koyama discovered polymyxins, initially, he had reported the colistin as a secondary metabolite of the Gram-positive soil bacterium *Paenibacillus polymyxa* subsp. *Colistinus* [12].

Historically, colistin was first used in the 1950s as an intravenous formulation. In 1959, the US FDA approved colistin as an antimicrobial agent against GNB due to its bactericidal activity for the treatment of various types of infections, including infectious diarrhoea and urinary tract infections. Moreover, polymyxins have been administered for several decades in topical formulations for eye and ear infections as well as for selective bowel decontamination. Additionally, polymyxins were used to fight infections caused by intractable GNB [7,13]. Colistin and polymyxin B have already been used for decades in veterinary medicine for prophylactic and therapeutic purposes [2].

Colistin is an active agent against aerobic Gram-negative pathogens that frequently represent the mainspring of life-threatening infections, such as carbapenem-resistant *P. aeruginosa*, *A. baumannii*, *K. pneumoniae, E. coli*, and other Enterobacteriaceae. Noteworthy, some bacterial species, such as; *Serratia marcescens*, *Proteus* spp., *Providencia* spp., *Morganella morganii, Vibrio cholera*, *Brucella*, *Campylobacter*, *Legionella*, *Chromobacterium*, *Neisseria* spp., *Edwardsiella* spp., some *Aeromonas* species, *Burkholderia cepacia*, anaerobic Gram-negative cocci, eukaryotic microbes, and mammalian cells, are possessing intrinsic colistin resistance [13,14].

**Figure 1.**
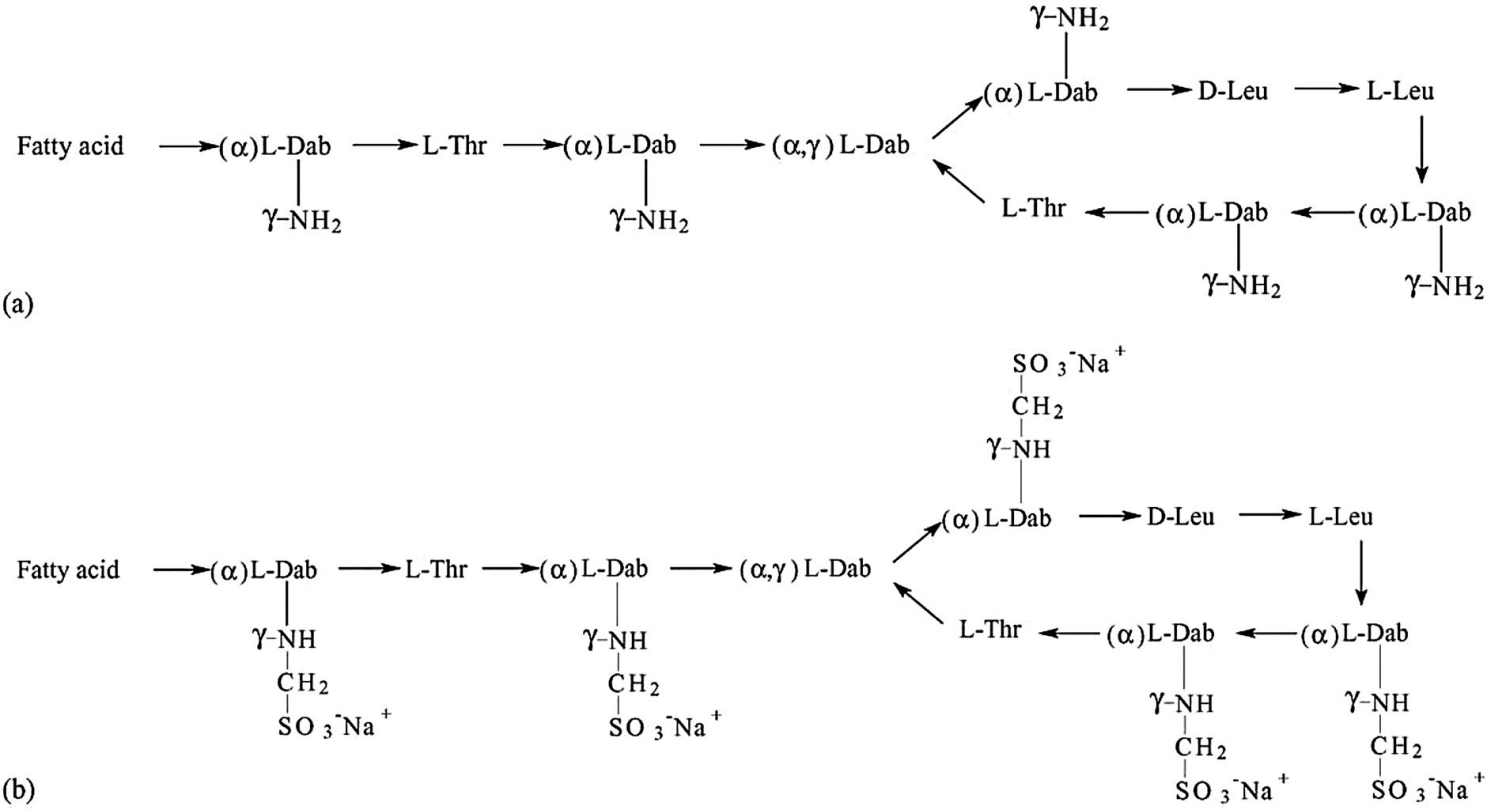
**(a) Structures of colistin A and B; (b) structures of sodium colistin A and B methanesulphonate.** Fatty acid: 6-methyl-octanoic acid for colistin A and 6-methyl-heptanoic acid for colistin B; Thr: threonine; Leu: leucine; Dab: α, γ-diaminobutyric acid. α and γ indicate the respective amino groups involved in the peptide linkage. Adapted from Li *et al*. [16].

In human medicine, two forms of colistin are clinically available for the treatment of infections caused by GNB, namely; colistin sulphate (CS) for oral and topical use; and the sodium salt of the negatively charged derivative of colistin known as colistin methanesulfonate (CMS) or colistimethate sodium (CMS), which is an inactive prodrug used for parenteral and nebulization formulations as it is less toxic than colistin sulphate (Figure 1). Among the two clinically available forms, colistin sulphate is the only form of colistin approved for use in pig production in some countries for the control of intestinal infections caused by Enterobacteriaceae, in particular, *E. coli* and *Salmonella* [4,15].

Regarding polymyxin B, although it exhibits a broad spectrum of activity, mostly against GNB, it has also been shown to be effective against Gram-positive bacteria such as; *Staphylococcus aureus* [17], *Streptococcus gordonii*, *Streptococcus agalactiae* [18], as well as against facultative anaerobic bacteria such as *Listeria monocytogenes* [19].

On account of the reported adverse events of polymyxins mainly nephrotoxicity and neurotoxicity, alongside to the discovery and approval of new and effective antibiotics, the clinical use of polymyxins was largely abandoned by the mid-1970s. However, they remained in clinical practice for patients suffering from cystic fibrosis (CF) due to pseudomonal lung infections and in topical solutions with other antimicrobial agents for the treatment of ear or eye infections [1,14,20].

By the mid-1990s the polymyxins had re-emerged as a last-resort treatment against MDR and XDR Gram-negatives, not because of an improved safety profile, but rather due to the emergence of XDR Gram-negative superbugs, particularly *P. aeruginosa*, *A. baumannii*, and *K. pneumonia*, which are resistant against all other available antibiotics, besides the lack of novel antimicrobials available to treat MDR bacterial infections [11,13].

Unfortunately, the overuse and misuse of colistin among humans and animals medicine have led to the global emergence of colistin-resistant pathogens. However, the development of bacteria resistant against colistin may also occur unaccompanied by any prior exposure to colistin, leaving clinicians barehanded to treat patients [18]. Indeed, the polymyxins now play a critical role mainly against life-threatening Gram-negative infections, as they are one of the few, and on occasion, the sole antimicrobial agent, retaining activity against MDR GNB [13].

**Figure 2.**
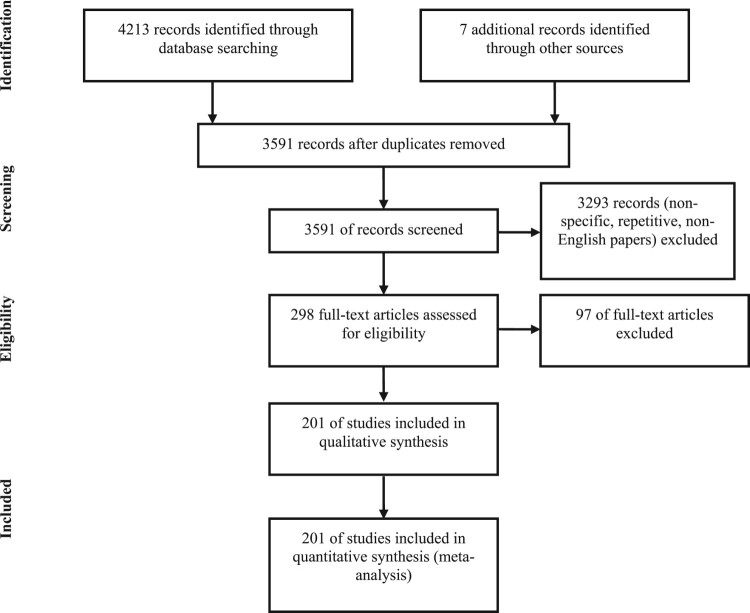
**PRISMA-modified flow diagram of included and excluded studies.** Adapted from the PRISMA website (http://www.prisma-statement.org/PRISMAStatement/FlowDiagram) and Liberati *et al*. [11].

Herein, we present an overview of the progression over the last two decades regarding the identification of alternative colistin mechanisms of action and different strategies taken by bacteria to develop resistance against colistin. To achieve this goal, we reviewed the published clinical data on colistin resistance among GNB, and a literature search was undertaken. MEDLINE (via Pub Med) and EMBASE were searched, limited by the dates 2000–2019, for articles using the following terms: [(colistin) AND (resistance OR resistant OR susceptible OR susceptibility)] OR (MCR genes). The results of this search were combined with separate searches for “Gram-negative bacteria” and “Enterobacteriaceae”. Other searches were also conducted on Pub Med regarding the *in-vitro* activity of colistin. Only articles published in English from 2000 onwards were collected in an attempt to include up to date relevant data. The PRISMA guidelines, according to Liberati *et al*. [21] were followed in searching, including, and excluding papers for this review (Figure 2).

## Mechanisms of antibacterial activity of polymyxins

As a result of the structural similarity between colistin and polymyxin B, it has been suggested that they share the same mechanisms of action [9,20]. The exact antibacterial mechanism by which colistin can kill bacterial cells is not well understood [13]. Colistin is mainly active against GNB due to the presence of lipopolysaccharide (LPS) in the GNB cell wall [10]. Therefore, understanding the outer membrane (OM) architecture of GNB is crucial to decipher the mechanism(s) of action of colistin.

One of the main functions of the OM is being a permeability barrier against various harmful agents, including different antimicrobials [6]. The protective role of the OM is mainly related to the presence of LPS in the surface of the cell that hinders the penetration of hydrophobic and/or large antibiotics via OM [8]. The structure of LPS comprises three domains: O antigen chain, a core polysaccharide, and a conserved lipid A that acts as a hydrophobic anchor in the OM [14]. The primary target of colistin is the LPS of the OM where it exerts its antibacterial action through direct interaction with the lipid A component of the LPS [6,15].

The saturated hydrocarbon chains of lipid A are enclosed together within the membrane by van der Waal forces, while the divalent magnesium (Mg^+2^) and calcium (Ca^+2^) cations associated with lipid A phosphoresters function to bridge adjacent LPS molecules, thus stabilizing the LPS molecules. The barrier function of the OM is further accentuated via the presence of the high negative charge carried on the lipid A phosphorester moieties, besides the phosphate and carboxylate groups within the core and O-antigen sugars [6].

### Colistin activity against Gram-negative bacteria

#### Direct antibacterial colistin activity

Generally, colistin kills bacteria by disrupting the bacterial outer and inner membranes via a long-accepted model, termed the “self-promoted uptake” pathway, which stated that the amphipathic nature of colistin is pivotal for the uptake of the colistin molecule across the OM barrier [13]. In this model, the initial fusion of colistin with the bacterial membrane occurs via electrostatic interactions between the cationic diaminobutyric acid (Dab) residues of colistin and anionic phosphate groups on the lipid A moiety of LPS in the OM of the GNB. Then, colistin competitively displaces the divalent cations Mg^+2^ and Ca^+2^, from the negatively charged phosphate groups of membrane lipids, destabilizing the LPS molecules, and weakening the membrane, thus permits the uptake of colistin. Thereafter, colistin attaches itself to the lipid A component of LPS, leading to derangement of the OM [1,16].

**Figure 3.**
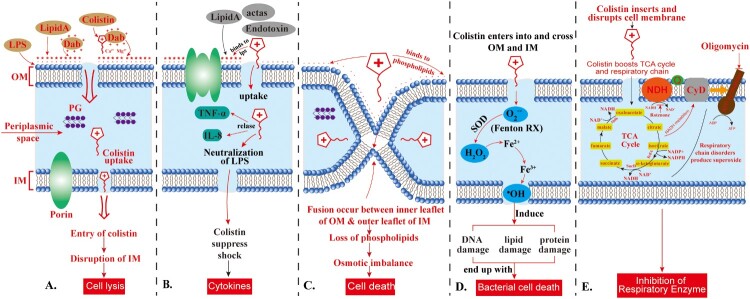
**Action of colistin on the Gram-negative bacterial membrane.** The cationic cyclic decapeptide structure of colistin binds with the anionic LPS molecules by displacing Mg^2+^ and Ca^2+^ from the outer cell membrane of Gram-negative bacteria, leading to permeability changes in the cell envelope and leakage of cell contents. LPS: lipopolysaccharides; PG: peptidoglycan; Dab: diaminobutyric acid (Dab); OM: outer membrane; IM: inner membrane. The scheme shows the five different mechanisms of antibacterial activity of colistin, namely; **(A)** Direct antibacterial colistin activity: the initial fusion of colistin with the bacterial membrane occurs via electrostatic interactions between the cationic diaminobutyric acid (Dab) residues of colistin and anionic phosphate groups on the lipid A moiety of LPS in the outer membrane, thus disrupting the bacterial outer and inner membranes and leads to cell lysis; **(B)** Anti-endotoxin colistin activity: The lipid A portion of LPS represents an endotoxin in Gram-negative bacteria. Thus, colistin inhibits the endotoxin activity of lipid A by binding to and neutralizing the LPS molecules, thus suppress the induction of shock through the release of cytokines such as tumour necrosis factor-alpha (TNF-α) and Interleukin 8 (IL-8); **(C)** Vesicle-Vesicle contact pathway: colistin bind to anionic phospholipid vesicles after transiting the OM leads to the fusion of the inner leaflet of the outer membrane with the outer leaflet of the cytoplasmic membrane, leading to loss of phospholipids and cell death; **(D)** Hydroxyl radical death pathway: Colistin acts via the production of the reactive oxygen species (ROS) this is known as, Fenton reaction, causing damage of DNA, lipid, and protein, and end up with cell death; and **(E)** Inhibition of respiratory enzymes: the antibacterial colistin activity is via the inhibition of the vital respiratory enzymes. Figure created using Adobe Illustrator version CC 2019 (23.1.0).

Noteworthy, the affinity of colistin for LPS is at least three times higher than its affinity for divalent cations [22]. This event leads to a detergent-like mechanism of action that involves an increase in the permeability of the cell envelope followed by leakage of cellular contents, and subsequently, colistin inserts its hydrophobic regions (fatty acyl tail and amino acids at positions 6 and 7) through these cracks in the OM resulting in “self-promoted uptake” [15], which leads to inner membrane lysis, leakage of periplasmic and cytoplasmic contents and ultimately cell death. Notably, this process is independent on the uptake of colistin into the cell [1,8,11,13,16,23] (Figure 3).

#### Vesicle-vesicle contact pathway

Another model for the antibacterial colistin activity is via an alternative mechanism called vesicle-vesicle contact, where colistin binds to anionic phospholipid vesicles after transiting the OM leading to the fusion of the inner leaflet of the OM with the outer leaflet of the cytoplasmic membrane, and thus promotes phospholipid exchange resulting in the loss of phospholipids. This event ends up with osmotic imbalance and lytic cell death [13,15] (Figure 3).

#### Hydroxyl radical death pathway

Colistin also acts through several other mechanisms, such as the hydroxyl radical death pathway via the production of the reactive oxygen species (ROS); hydroxyl radicals (^•^OH), superoxide (O_2_^-^), and hydrogen peroxide (H_2_O_2_), which cause oxidative stress. Generally, O_2_^-^ is generated when colistin enters into and crosses the OM and IM. This is followed by the conversion of O_2_^-^ into H_2_O_2_ by superoxide dismutase (SOD). Then, H_2_O_2_ oxidizes ferrous iron (Fe^2+^) into ferric iron (Fe^3+^), besides the formation of **^·^**OH, this process is known as Fenton reaction. This reaction can induce oxidative damage in bacterial DNA, proteins, and lipids, which ultimately lead to cell death.

Of note, during this reaction, damage and resynthesis of Fe-S dependent proteins, especially Fe-S dependent dehydratase, such as dihydroxy-acid dehydratase (DHAD), take place, where the exposed Fe-S cluster is damaged by one of two ways; oxidation by O_2_^-^ to an unstable species with the formation of H_2_O_2_ and release of Fe^2+^ ions or oxidation by H_2_O_2_, leading to the loss of Fe^3+^ and inactivation of Fe-S dependent protein. Then the inactive Fe-S cluster can be restored by YggX (a protein member of the SoxRS regulon) and a di-iron protein YtfE in the presence of Fe^3+^ ions. This mechanism of killing has been shown to occur in the polymyxin-sensitive and MDR isolates of *A. baumannii* and *E. coli* but does not take place in polymyxin-resistant strains [22,24] (Figure 3).

#### Inhibition of respiratory enzymes

A secondary mechanism for the antibacterial colistin activity is via the inhibition of the vital respiratory enzymes. Generally, the bacterial respiratory chain is composed of three complexes with quinones and reduced nicotinamide adenine dinucleotide (NADH), which act as the carriers that shuttle electrons and protons between large protein complexes. In the case of complex 1, three different inner membrane respiratory enzymes of the NADH oxidase family, namely; proton-translocating NADH-quinone (Q) oxidoreductase (NADH-1), NADH-Q oxidoreductase that lacks an energy-coupling site (NADH-2), and the sodium-translocating NADH-Q oxidoreductase have been identified.

The inhibition of NADH oxidase enzymes by colistin has been reported in Gram-positive *Bacillus* spp. [25], while in *Mycobacterium smegmatis* the inhibition of an alternative NADH-dehydrogenase and malate:quinone oxidoreductase by colistin has been reported [26]. Generally, this mechanism has been described in *E. coli*, *K. pneumoniae*, and *A*. *baumannii* [20,23,27] (Figure 3).

#### Anti-endotoxin colistin activity

Besides the direct antibacterial activity, colistin also exerts potent anti-endotoxin activity, where the lipid A portion of LPS represents an endotoxin in GNB. Therefore, colistin inhibits the endotoxin activity of lipid A by binding to and neutralizing the LPS molecules. The significance of this mechanism for the *in vivo* antibacterial activity is via the suppression of the endotoxin's ability to induce shock through the release of cytokines such as tumour necrosis factor-alpha (TNF-α) and Interleukin 8 (IL-8) (Figure 3). Indeed, this suppressing action is still not clear, since the plasma endotoxin immediately bounds by LPS-binding protein, and the complex is quickly bound to cell-surface CD14 [1,28].

### Polymyxins activity against Gram-positive bacteria

Generally, polymyxins display reduced activity against Gram-positive bacteria as they do not attach favourably to lipoteichoic acid found in the cytoplasmic membrane. However, as previously mentioned, polymyxin B has been found to have a broad spectrum of activity, mostly against GNB, but has also been shown to be effective against Gram-positive bacteria [17,18].

Indeed, the mechanism of action of polymyxin B is not based on a detergent or lytic effect on the bacterial membrane, as it has been previously reported for colistin. It has been demonstrated that polymyxin B induces the apposition of anionic vesicles in addition to the formation of functional vesicle-vesicle contacts that permit a fast and selective exchange of phospholipids particularly between the outer monolayers of the vesicles [29]. Of note, the insertion of hydrophobic functional groups to the structure of polymyxin B through the acylation of the amine side-chain of Dab1 with different fatty acids may increase its ability to enter across membranes and confer favourable interactions with lipoteichoic acid of Gram-positive bacterial membranes, leading to enhanced antibacterial activity [30].

Very recently, Yu *et al*. [25] have revealed that colistin can induce ROS accumulation in Gram-positive bacteria, including *Paenibacillus polymyxa C12*, *Bacillus subtilis* WB800, and *P. polymyxa* ATCC842, leading to oxidative stress regardless of cell membrane lysis, which results in cell death. The generation of oxidative stress is related to the sequenced stimulation of the tricarboxylic acid (TCA) cycle and respiratory chain, followed by the transient depletion of NADH. Indeed, the detailed mechanism of oxidative stress formation by colistin is still not fully elucidated [25].

## Polymyxin derivatives act as potentiators to sensitize GNB towards other antibiotics

Polymyxin B nonapeptide (PMBN) is a deacylated derivative of polymyxin B, which lacks the fatty acyl tail and the Dab residue at position 1. Therefore, PMBN exerts virtually no antibacterial activity as polymyxin B; however, PMBN still exerts the endotoxin-binding activity as polymyxin B by interacting with the anionic moieties of LPS, although this effect is less active than that of polymyxin B. Additionally, the disorganizing effect in the OM caused by PMBN enhances the permeability of the bacterial membrane to hydrophobic antibiotics [31]. Thus, it acts as permeabiliser, sensitizer or potentiator, expanding the spectrum of activity of numerous anti-Gram-positive antibiotics to include the GNB to able to defeat GNB infections even at low concentrations (1–3 mg/L) [32].

Moreover, it has been reported that several antibiotics, when used in combination with colistin, lead to growth-inhibition at levels below their corresponding clinical breakpoints. In case of colistin-resistant strains of Enterobacteriaceae expressing plasmid-borne *mcr-1*, the administration of clinically relevant concentrations of colistin in combination with other antibiotics that are formerly inactive against GNB but are typically active against Gram-positive bacteria will induce their antibacterial activity [33]. For instance, the combination therapy of colistin with clarithromycin shows efficacy against *mcr-1*-positive *K. pneumoniae* in murine thigh and bacteremia infection models at clinically relevant doses. This indicated that this combination could represent a vital therapeutic choice against highly drug-resistant GNB expressing *mcr-1* [33].

## Overview of mechanisms underlying polymyxin resistance

### Chromosomally encoded resistance to colistin

The mechanisms underlying polymyxins resistance in GNB are complex and not completely understood until now [5]. Generally, GNB can develop resistance to polymyxins through intrinsic, mutation or adaptation mechanisms, besides the horizontally acquired resistance mediated via the *mcr-1* gene and its variants [34,35]. Cross-resistance between colistin and polymyxin B has been reported [1,16]. Although the underlying mechanisms of resistance are common among GNB, they may differ between different species [14,36].

Herein, we aimed to give an overview of the current situation regarding polymyxins resistance, focusing mainly on colistin resistance (Table 1). The main polymyxins resistance mechanisms can be summarized as follows: (i) modifications of the LPS moiety via the addition of cationic groups to the LPS; (ii) mutations that lead to the loss of the LPS; (iii) porin mutations and overexpression of efflux pump systems; (iv) overproduction of capsular polysaccharide (CPS) in some GNB that hide the polymyxin binding sites and the release of CPS trapping polymyxins; and (v) enzymatic inactivation of colistin [23].

**Table 1.** Characteristics of mechanisms of resistance and modifications associated with polymyxin resistance.

Bacteria	Resistance mechanisms	Modifications	Genes / involved determinants	References
*K. pneumoniae*	Modifications of the LPS moiety Overproduction of capsular polysaccharide Efflux pump systems Membrane fluidity/permeability	L-Ara4N and/or PEtN modification of lipid A Overexpression of *phoPQ* operon Overexpression of *pmrAB* operon Overproduction of CPS Multi-drug efflux pump Regulate the permeability barriers of the bacterial outer membrane	*pmrA, pmrB, phoP, phoQ, eptB mgrB* (also known as y*obG*) *ccrB siaD, OmpA, cps* operon (*wca*) *kpnEF, acrAB, yrbB-F, oqxAB* The regulator RamA	[13,37,38] [23,38] [39] [18,23,40,41] [5,18,23,40,42,43] [5]
*A. baumannii*	Modifications of the LPS moiety Loss of LPS Membrane fluidity/permeability Efflux pump systems Other polymyxin resistance mechanism Unclear	Related to the L-Ara4N biosynthesis Influencing the operon *pmrCAB* expression Deacylation of lipid A Inactivation of lipid A biosynthesis Abolishing LPS synthesis Alteration in membrane composition Efflux pump Decreasing biotin synthesis Detoxifying reactive oxygen insert in a mobile genetic element	*pmrF operon pmrA, pmrB, pmrC naxD lpxA, lpxC, lpxD, lptD lpsB vacJ adeABC,* HlyD family*, emrA, emrB* Genes related to biotin synthesis *sodB, sodC* A duplicated ISAbaI-*eptA* cassette	[3] [42,44–46] [3] [3,5,23,34,47,48] [34,49] [50] [46,51] [34] [18] [52]
*P. aeruginosa*	Modifications of the LPS moiety Loss of LPS Efflux pump systems Unclear	LPS additions in response to high Zn^2+^ LPS additions in response to high Zn^2+^, multidrug efflux pump LPS additions in response to low Zn^2+^ Activation of the two-component system (TCS) Inactivation of lipid A biosynthesis Multidrug efflux pump Unclear	*colR/colS, cprRS parR/parS* The protein OprH or H1 *pmrA, pmrB, phoP, phoQ lpxC, lpxO2 rsmA, parR/parS PA1199/2583/5548/2928/1980/5447/4541/1938*	[13,23] [53] [16,18,40] [23,40,54] [5] [18,55] [56]
*S. enterica*	Modifications of the LPS moiety Membrane fluidity/permeability	L-Ara4N and/or PEtN modification of lipid A Deacylation of lipid A, stimulating the transcription of genes in adaptation Activation of the two-component system (TCS) Alterations in membrane composition	*arnBCADTEF pagL, rpoN pmrA, pmrB, phoP, phoQ ompD*	[22,36] [36] [23] [23]
***Helicobacter pylori***	Modifications of the LPS moiety	Modification of lipid A	*Cgt*	[23]
*V. cholera*	Modifications of the LPS moiety	Linked to LPS biosynthesis and modification	*gspIEF, lpxN, vc0224/0239/1981*	[18]
***Haemophilus influenza***	Other polymyxin resistance mechanism	Involved in LOS biosynthesis	*lic1/2A, lpsA, lgtF, opsX*	[57]
***Burkholderia multivorans***	Membrane fluidity/permeability	implicated in stabilizing OM permeability	*buml_2133/2134*	[18]

The primary strategy that allows GNB to escape the bactericidal effect of polymyxins depends on performing alterations in the LPSs of the GNB-OM, mainly by reducing the negative charge of the OM, thus hinders the binding and the action of colistin [3,23,58]. This strategy can be achieved by replacing the phosphate groups of lipid A by the cationic 4-amino-4-deoxy-L-arabinose (L-Ara4N) and/or phosphoethanolamine (PEtN) moieties [13,22]. This can be accomplished mostly via two-component regulatory systems (TCSs) [36,44]. Mutations in these regulatory systems or their regulators lead to their upregulation that is accompanied by the addition of more cationic moieties to LPS, which in turn, decreases the net negative charge of the OM, and preventing the action of colistin [3].

Noteworthy, the modification of the L-Ara4N moiety is more effective than that of the PEtN moiety because the reduction in the net negative charge leads to colistin resistance more efficiently with the L-Ara4N moiety [58]. In this context, it has been reported that L-Ara4N modification reduces the net anionic charge of lipid A to 0, while the PEtN modification decreases it from -1.5 to −1 [59,60].

Two of the most extensively studied TCSs are the PhoPQ and PmrAB systems whose functions and regulations have been found to overlap. Different genes that encode the LPS-modifying enzymes include; i) the *pmrCAB* operon system that encodes for three functional proteins, namely; pEtN phosphotransferase PmrC (also known as *eptA*), the response regulator PmrA (also known as BasR), and the sensor kinase protein PmrB (also known as BasS). The function of the pEtN phosphotransferase PmrC is the addition of the cationic pEtN moiety to the lipid A of LPS [4,23,42].

PmrB is a protein owning tyrosine kinase activity that activates PmrA through phosphorylation. PmrA then activates the transcription of the *pmrCAB* operon, the *pmrHFIJKLM* operon (also called the *arnBCADTEF* or *pbgPE* operon), and the *pmrE* gene involved in LPS modification (pEtN and L-Ara4N addition to LPS). ii) The *pmrHFIJKLM* operon and the *pmrE* gene are responsible for the synthesis of the L-Ara4N moiety and its binding to lipid A [4].

PhoP/PhoQ and PmrA/PmrB TCSs both contain a sensor kinase; PhoQ and PmrB, respectively, which can senses the environmental signals, such as the reduction in cell envelope Mg^+2^ and Ca^+2^ contents and low pH, besides the presence of colistin, thus they can change the expression patterns of these TCSs [4,42].

The activation of PhoQ and PmrB leads to the phosphorylation of the response regulators; PhoP and PmrA, respectively. This phosphorylation, in turn, enhances the binding of these regulators to the promoters of regulated genes. The phosphorylation of PhoP increases the transcription of several genes, including *pmrD*, whose product binds to and stabilizes PmrA in its phosphorylated state [6,11].

Other modifications related to chromosomally polymyxin resistance have been reported, such as the decrease in the number of acyl moieties via *lpxR*-like deacylation and hydroxylation of lipid A [58]. Besides, acylation of lipid A, these modifications are capable of changing the permeability barrier properties of the OM [18].

## Examples of chromosomally encoding colistin resistance among MDR GNB

***K. pneumoniae***

In *K*. *pneumoniae*, the polymyxins resistance is mediated by different strategies such as; the modification of lipid A via mutations in *pmrA*, *pmrB* or *phoQ* genes, which in turn, upregulate the PhoP/PhoQ and PmrA/PmrB systems [60], leading to the addition of either L-Ara4N or PEtN to LPS [13]. Besides, mutations in the *mgrB* gene (a negative feedback regulator of the PhoPQ system) that encodes the MgrB protein (also known as YobG); a small regulatory transmembrane protein composed of 47 amino acids. The *mgrB* gene is upregulated upon activation of the PhoP system. The MgrB protein, in turn, suppresses the expression of the PhoQ-encoding gene, *eptB*, leading to negative regulation of the kinase activity of PhoQ and decreased PEtN production [3,37,38]. In this context, it has been reported that one of the genes negatively regulated by PhoQ/PhoP, via MgrB, is *eptB*, also involved in LPS modification, which can add PEtN to different sites of LPS. This *eptB* encodes a phosphoethanolamine transferase, which modifies LPS at the outer 3-deoxy-D-manno-octulosonic acid (Kdo) residue with phosphoethanolamine. The addition of a pEtN moiety to the Kdo residue of LPS decreases the net negative charge of molecules and reduces the electrostatic repulsion between neighbouring LPS molecules, thus leading to polymyxin resistance [61].

While the inactivation or deletion of the *mgrB* gene causes the overexpression of the *phoPQ* operon that in turn, activates the *arnBCADTEF* operon leading to L-Ara4N biosynthesis, and thus increases colistin resistance [23,38]. In this context, it has been previously reported that insertional inactivation of the *mgrB* gene, encoding a negative-feedback regulator of the PhoQ-PhoP signalling system, can be responsible for the acquired colistin resistance in *Klebsiella pneumoniae* strains producing KPC-type carbapenemases (KPC-KP), by upregulating PhoQ-PhoP system, which, in turn, upregulates the Pmr lipopolysaccharide modification system responsible for modification of the lipopolysaccharide polymyxin target [62]. The *mgrB* gene has been detected in colistin-resistant *K. pneumoniae* and *K. oxytoca* [5].

Mutations of the *mgrB* gene by insertion sequences (IS5-like, IS1F, ISKpn13, ISKpn14, IS10R) or point mutations represent the primary cause of polymyxin resistance in clinical *K*. *pneumoniae* strains [37,39].

Recently, mutations in the *ccrB* (colistin resistance regulation) operon have been described. This operon codes for two proteins, namely; the regulatory protein CrrA and the sensor protein kinase CrrB [39]. In *K. pneumoniae*, it has been reported that the inactivation of the *crrB* gene leads to the overexpression of the *pmrAB* operon that in turn, leads to activation of the *pmrHFIJKLM* operon, *pmrC*, and *pmrE* genes, which ends up with the addition of L-Ara4N and pEtN to the lipid A of LPS [39].

Another polymyxin resistance mechanism in *K*. *pneumoniae* is the overproduction of the surface anionic capsular polysaccharides (CPS) [23] that represent a protective barrier against polymyxins, where the upregulation of capsular biosynthesis genes, namely; *siaD*, *OmpA*, and *cps* operon (*wca*), hinders the binding of polymyxins with lipid A [18] by trapping polymyxins [40]. Fresno *et al*. [41] reported that the association between the surface CPS and the LPS is mediated through an ionic interaction that is stabilized by divalent cations. Therefore, the presence of polymyxins, which disturb the cation-dependent bridges between the molecules of LPS results in the release of CPS.

Moreover, it has been reported that the acylation of lipid A in *K. pneumoniae* could be regulated by *lpxM* (formally *msbB* or *waaN*), where its inactivation can lead to a lack of the L-Ara4N modification along with a subsequent reduction in polymyxin resistance [63].

***A. baumannii***

Adams *et al*. [45] showed the first evidence that the modification of lipid A structure by the addition of pEtN to LPS is associated with mutations in *pmrA* and *pmrB* genes in *A. baumannii*. Several studies have revealed that colistin-resistant *A. baumannii* isolates could change back into a susceptible phenotype through mutations in PmrA/B, which in turn, downregulates the operon PmrCAB expression [44–46]. Additionally, it has been reported that the partial removal of *pmrC* is associated with an increase in the susceptibility of colistin-resistant *A. baumannii* [42].

Noteworthy, *A. baumannii*, in contrast with other Enterobacteriaceae, is devoid of all required genes for L-Ara4N biosynthesis due to the absence of the *arn* operon that is responsible for the expression of the enzymes implicated in L-Ara4N biosynthesis [3].

The expression of *naxD*, a gene encoding the enzyme deacetylase that is necessary for the conversion of N-acetylgalactosamine to galactosamine before its binding to lipid A, is dependent on the activation of PmrB [3]. It has been shown that low to moderate colistin resistance levels can be achieved in *A. baumannii* via the binding of galactosamine to the 1′-phosphate position of lipid A, upon activation of the sensor kinase PmrB [13].

Moreover, mutations in the *lpxA*, *lpxC*, and *lpxD* genes of *A. baumannii* lead to the inactivation of lipid A biosynthesis; thus, a complete loss of LPS occurs with subsequent loss of the polymyxin target and consequently results in very high colistin minimum inhibitory concentrations (MICs) (128 mg/L) [3,5,34], this effect has also been confirmed by Moffatt *et al*. [47] who demonstrated that full inactivation of the genes related to lipid A biosynthesis *(lpxA*, *lpxC* or *lpxD*) leads to the complete loss of surface LPS in *A. baumannii*.

Mutations detected in those genes were found to be mediated either by substitutions, truncations, frameshifts or insertional inactivation via the insertion sequence ISAba11 [23].

Additionally, *lptD*, *lpsB*, *vacJ*, and the locus of biotin synthesis, were identified in *A. baumannii* as contributors for polymyxins resistance. LptD (essential for the insertion of the newly synthesized LPS into the OM), was found implicated in polymyxins resistance in *A. baumannii* [42,48]. Bojkovic *et al*. [48] reported that the removal of *lptD* results in the complete loss of LPS and reduction in polymyxin resistance in *A. baumannii*, whereas the *lpsB* gene protects *A. baumannii* from the bactericidal effect of colistin via encoding the glycosyltransferase that is responsible for the LPS synthesis [49]. Nhu *et al*. [50] revealed that a single mutation in *vacJ* (R166N) of *A. baumannii* contributes to a highly colistin-resistant phenotype.

Biotin is an essential co-factor of lipid metabolism has been documented as a crucial factor related to the sensitivity of the polymyxins in *A. baumannii*, where higher biotin levels lead to an increase in the production of lipid A with a subsequent increase in colistin sensitivity [49]. Hood *et al*. [34] revealed that the removal of genes related to biotin synthesis results in the reduction of the susceptibility of *A. baumannii* to colistin.

Furthermore, it has been reported that the *sodB* (A1S_2343) and *sodC* genes mediate colistin resistance, most likely by detoxifying reactive oxygen species in *A. baumannii* [18].

Very recently, Trebosc *et al*. [52] showed the novel colistin resistance mechanism of *A. baumannii* mediated by genetic integration of the insertion element ISAbaI upstream of the PmrC homolog EptA (93% identity), leading to its overexpression. Besides, the detection of a duplicated ISAbaI-eptA cassette, suggesting that this colistin resistance determinant may be inserted in a mobile genetic element.

***P. aeruginosa***

Similarly to *A. baumannii*, mutations in the *lpxC* or *lpxO2* genes of *P*. *aeruginosa* lead to the inactivation of lipid A biosynthesis, resulting in loss of LPS from the outer cell wall with the subsequent loss of the polymyxin target [5].

In *P. aeruginosa*, polymyxin resistance is mediated by the addition of L-Ara4N to the phosphate groups in lipid A of LPS via the *arn* (*pmr*) operon that is upregulated by PmrA/PmrB and PhoP/PhoQ TCSs [54]. Three other TCSs have been implicated in colistin resistance in *P. aeruginosa*, namely; ColR/ColS, ParR/ParS, and CprRS [23]. The ColR/ColS TCS is upregulated in the presence of an excess of extracellular Zn^2+^, leading to the addition of PEtN to lipid A with subsequent colistin resistance [13].

Several studies have reported that mutations in PmrB, PhoQ, ParR, and ParS proteins in clinical *P. aeruginosa* isolates, cause the constitutive overexpression of the LPS modification operon *pmrHFIJKLM* via the activation of one of the components of the TCSs (PmrB, ParS, ParR) or the inactivation of sensor kinase PhoQ, which acts as a repressor of PhoP activity, thus allowing the PhoP to stimulate *pmrHFIJKLM* operon expression, leading to the addition of L -Ara4N to the LPS, which, in turn, causes different degrees of colistin resistance [40,54].

Noteworthy, the occurrence of mutations in the *phoQ* gene and the *colS* or *cprS* gene simultaneously, permits a high level of colistin resistance. The action of the ColRS and CprRS TCSs was suggested to occur via the stimulation of the *phoQ* gene [23].

A previous study has reported that the presence of extracellular DNA in *P. aeruginosa* is associated with colistin and polymyxin B resistance through the activation of PhoPQ and PmrAB systems [23].

Furthermore, in the presence of reduced levels of the cell envelope Mg^2+^, the outer membrane protein OprH (or H1) is overexpressed and binds to the negatively charged phosphate groups, and thus hinders the polymyxin binding and develops polymyxin resistance in *P. aeruginosa* [16,18,40]. Besides, it has been reported that the down-regulation of porin (OprD) can affect the polymyxins resistance in *P. aeruginosa* [18]. Perez *et al*. [40] revealed that polymyxin resistance could also occur via the trapping of polymyxins in the bacterial capsule of *P. aeruginosa*.

Intriguingly, the role of some genes, such as; PA1199, PA2583, PA5548, PA2928 (genes that most likely contribute to LPS biosynthesis), PA1980 (*eraR*), PA5447 (*wbpZ*), PA4541, and PA1938 (non-LPS-mediated genes), in mediating colistin resistance in *P. aeruginosa*, is still unclear [56].

***Salmonella enterica***

In *S. enterica*, colistin resistance is mediated via the activation of the PmrA/PmrB and PhoP/PhoQ TCSs, by various environmental stimuli, such as low concentrations of Mg^2+^ or with specific mutations in the TCSs-encoding genes, which in turn, activate the *arnBCADTEF* and *pmrCAB* operons, thus leads to the biosynthesis and addition of L-Ara4N and PEtN, respectively, to lipid A [22,36].

Other modifications mediating colistin resistance in *S. enterica*, include the deacylation of lipid A by PagL. Additionally, RpoN stimulates the transcription of genes implicated in the adaptation and survival of bacterial cells; however, these mechanisms are less common in *S. enterica* [36].

It has been reported that a periplasmic protein (YdeI) regulated by the PhoPQ and PmrAB TCSs, can associate with the OmpD porin and consequently, increases the bacterial resistance to polymyxins in *S. enterica* [23].

In *S. Typhimurium*, it has been reported that the acylation of lipid A can be regulated by *lpxM*, where its inactivation can lead to a lack in the L-Ara4N modification, and thus decrease the polymyxin resistance [18].

It has been reported that the presence of extracellular DNA in *S. Typhimurium* is associated with colistin and polymyxin B resistance through the activation of PhoPQ and PmrAB systems [23].

## Miscellaneous examples for chromosomally encoded colistin resistance

Noteworthy, the *cgt* gene, which plays a role in lipid A modification, has been shown to mediate colistin resistance in *Helicobacter pylori* [23].

In *V. cholera*, it has been reported that different genes linked with type II secretion system, namely; *vc2728* (*gspI*), *vc2731* (*gspF*), *vc2732* (*gspE*), *vc0212* (*lpxN*), *vc0224*, *vc0239*, and *vc1981*, are implicated in the biosynthesis and modification of LPS, and thus contribute to polymyxin resistance. Besides, the acylation of lipid A could be regulated by *lpxM*, in which its inactivation can lead to a lack in the L-Ara4N modification with the subsequent reduction in polymyxin resistance [18].

Mutations in *lic1*, *lic2A*, *lpsA*, *lgtF*, and *opsX* genes that are involved in lipooligosaccharide (LOS) biosynthesis, have been shown to contribute to reducing the polymyxin resistance in *Haemophilus influenza* [57].

In *Burkholderia multivorans*, it has been reported that the putative hopanoid biosynthesis genes, namely; Bmul_2133 and Bmul_2134, have been implicated in the stabilization of OM permeability, thus contribute to polymyxin resistance through a mechanism that is independent of LPS-binding activity.

Additionally, other genes such as *suhB Bc*, *bvrR/S* TCSs, *epsC-N*, *cgh* (choloylglycine hydrolase), *waaL*, *rfbA*, *vacJ*, and *ompW*, have been implicated in polymyxin B or colistin resistance in various bacterial strains, via modifications in OM composition [18].

## Heteroresistance

Phenotypically, polymyxins resistance can be acquired through polymyxin-heteroresistant bacteria. These bacteria yield subpopulations with different degrees of susceptibility to polymyxins. The MICs of polymyxins in these bacteria are ≤ 2 mg/l; however, the subpopulations can survive in the presence of > 2 mg/l polymyxins, which in turn, results in the amplification of the resistant subpopulations in the presence of polymyxin alone and consequently, develop polymyxin resistance. Although the frequency of polymyxin heteroresistance in *P. aeruginosa* is scarce, it is more frequently detected among MDR *A. baumannii* and *K. pneumonia* [13].

The detection of heteroresistance can be performed using microdilution assays, where the heteroresistant bacteria display a “skipped wells” phenomenon (wells with no growth, although growth still occurs at higher concentrations) [3]. Besides, Gefen *et al*. [64] have presented a novel method named “TDtest”, which is a modification of the standard disk-diffusion assay. It allows the detection of tolerant and persistent bacteria by enhancing the growth of the surviving bacteria inside the inhibition zone, once the antimicrobial agent has diffused away.

At a mechanical level, heteroresistance to polymyxins was suggested to be due to mutations in chromosomal genes, such as lipid A biosynthesis genes (*lpxA*, *lpxC*, *lpxD*) or the addition of L-ara4N, which in turn, affect the response regulator PhoP. Several studies have proved that the mechanism of heteroresistance confers a high level of resistance (MIC >128 mg/L) [44,47].

Snitkin *et al*. [44] revealed that although the mutant heteroresistant strains are stable, the original susceptible isolate may be able to re-emerge in some patients. This phenomenon may be attributed to the occurrence of dormant persisters or due to the presence of bacteria in non-accessible sites by polymyxins [20].

Interestingly, Herrera *et al*. [65] reported that at mildly acidic pH (5.8), a strong induction of the addition of L-Ara4N and PEtN takes place, which in turn, contributes to polymyxin resistance. Similarly, it has been reported that polymyxin resistance is attributed to the acidic growth conditions of bacteria via the transcriptional activity of several genes, including; *yjdB*, *pmrC*, *pagB*, and *pmrF* [18].

## Efflux pumps

Since polymyxins have an amphipathic nature and act likewise as other biological detergents, therefore, the efflux pump system may be involved in their resistance [42]. Commonly, the activation of these pumps results in an increase in resistance to different antibiotics concurrently, including colistin. In various bacterial species, different efflux pumps, such as Sap (sensitive antimicrobial peptides) proteins, BrlR, the AcrAB-TolC complex or KpnEF, have been reported. Sap proteins are composed of five different proteins encoded by the *sapABCDF* operon [60]. In various organisms, the AcrAB-TolC, KpnEF, MtrC-MtrD-MtrE, VexAB, RosAB, and NorM efflux pumps have been designated to give tolerance toward polymyxin B [66].

In *K. pneumoniae*, polymyxin resistance can also occur via the activation of the efflux pumps AcrAB [23,40]. Indeed, efflux pump systems associated with colistin resistance have not been well studied. However, it has been reported that mutations in kpnEF and AcrAB, encoding components of efflux pumps, can decrease the MIC of colistin (2-fold) and enhance the survival of bacteria at low concentrations of polymyxin [23].

The role of kpnEF in capsular synthesis has been confirmed through a multi-drug efflux pump kpnEF mutant that showed a defect in capsular synthesis [18]. Trimble *et al*. [66] showed that the overexpression of the intrinsic regulator, RamA, in *K. pneumoniae* increased polymyxin B resistance through different mechanisms, including the modulation of efflux pump genes such as *acrAB*, *yrbB-F*, and *oqxAB*.

It is worth mentioning that the efflux transporter protein families, AdeABC and HlyD, in *A. baumannii* have been shown to contribute to polymyxin resistance [46]. In this context, the role of an efflux system in the induction of polymyxin resistance in *A. baumannii* was demonstrated by Lin *et al.* [51] who divulged by searching a genome database, the presence of four pairs of *emr*-like genes (transporter proteins) namely, *emrB* and *emrA* genes. Deletion of the *emrB* gene resulted in perturbation of the ability to pump out, confirming its role as an efflux pump like Emr transporters with subsequent increase in the susceptibility of *A. baumannii* towards colistin [51].

In *A. baumannii*, eighteen putative efflux transporters were found upregulated in response to the physiological level of NaCl, resulting in increased tolerance to various antibiotics, including colistin [67].

Moreover, Muller *et al*. [53] demonstrated that mutations in ParR and ParS proteins in *P. aeruginosa* could also contribute to enhancing the production of the multidrug efflux system MexXY/OprM mechanism that provides low to moderate resistance levels to polymyxins. In this context, RsmA, a small RNA-binding protein, has been shown to contribute to polymyxin B and colistin resistance via its role in the type three secretion system (TTSS) in *P. aeruginosa* [18].

Otherwise, the multidrug efflux pump activator, BrlR, a member of the MerR family that is present in *P. aeruginosa*, can bind to the *oprH* promoter of the *oprH-phoPQ* operon and downregulates the *phoPQ* TCS, leading to increased colistin susceptibility via reduced transcription of the *pmrAB* and *arnT* systems [68].

Furthermore, Da Silva *et al*. [67] revealed that the use of efflux inhibitors, such as carbonyl cyanide 3-chlorophenylhydrazone (CCCP) could decrease the resistance pattern of colistin in *A. baumannii*, *K. pneumoniae*, *P. aeruginosa*, and *Stenotrophomonas maltophilia*, strongly suggesting the involvement of efflux pumps in the colistin resistance phenotype.

### Plasmid-mediated resistance to polymyxins

In addition to the mutations-based mechanisms of resistance mentioned above, the horizontal transfer of a plasmid-borne gene; *mcr-1* (standing for mobile colistin resistance) has become a significant cause for the dissemination of polymyxin resistance among various GNB [3,4,35]. Indeed, the emanation of the MCR enzymes could be tracked down to the 1980s in China and 2005 in France, in pathogens isolated from poultry and veal calves, respectively [69].

In late 2015, the plasmid-mediated *mcr-1* gene was first described in an *E. coli* strain isolated from food animals in China [35]. Since then, dissemination of *mcr-1* among different Enterobacteriaceae strains, including *E. coli*, *K. pneumoniae*, *Enterobacter cloacae*, *Enterobacter aerogenes*, *Cronobacter sakazakii*, *S. enterica*, *Raoultella ornithinolytica*, *Citrobacter freundii*, *Citrobacter braakii*, *Shigella sonnei*, *Kluyvera ascorbata*, and *Moraxella* spp. [3,43] has been reported worldwide in over 30 countries across five continents [2,36,43,70] in farm and wild animals, food (meat and vegetables), humans (colonized and infected), aquatic environments [3,55,58], hospital sewage [55], wild birds [71,72], and vector insects (housefly/blowfly). Although several reports have proposed that flies may serve as intermediate vectors for the transmission of *mcr-1* between animals and humans, the exact path for the circulation/spread of *mcr-1* remains ambiguous [43].

This global dissemination of the *mcr-1* gene suggests that the use of colistin in veterinary medicine has probably sped up that dissemination among animals and humans, and this is consistent with the hypothesis that livestock, primarily pigs are most likely the primary source of MCR-1 producers [36].

**Figure 4.**
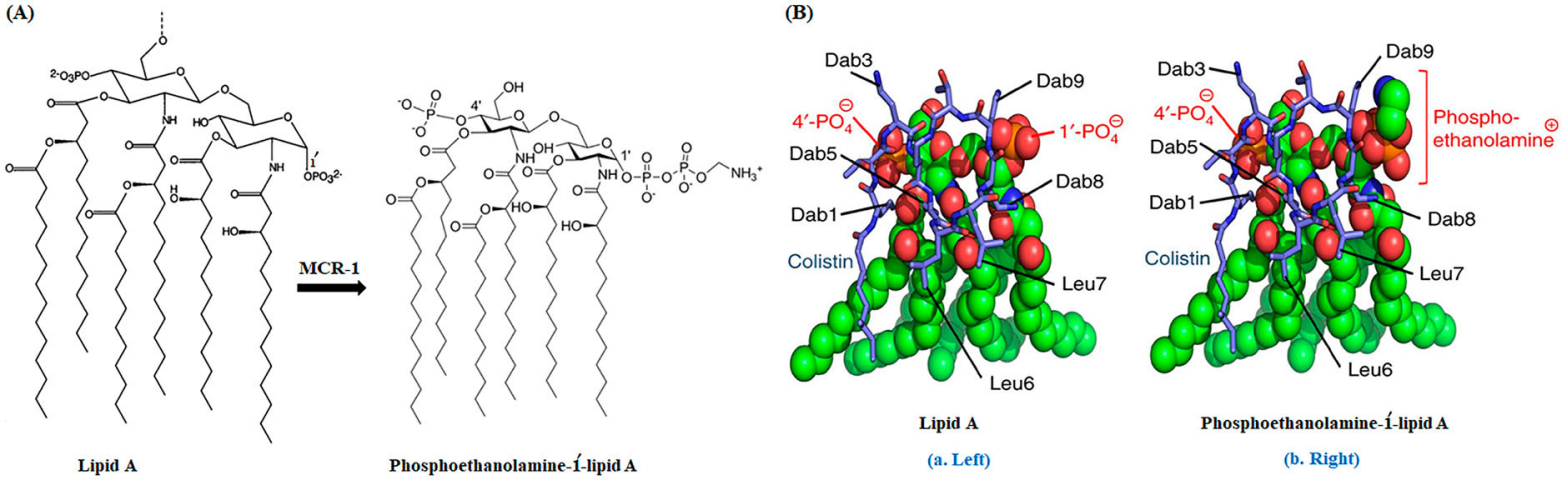
**Scheme of colistin binding to lipid A. (A)** a Schematic of the transfer of phosphoethanolamine to the 1-PO4 group of Hexa-acylated lipid A in the presence of MCR-1. **(B)** Models of colistin (blue sticks) binding to lipid A (left) or phosphoethanolamine-1΄-lipid A (right) (spheres coloured green, red, blue, and orange for C, O, N, and P atoms, respectively). **a (left)**, The positively charged Dab colistin residues interact with the negatively-charged 1′ and 4′ phosphate groups of lipid A, reducing the net-negative charge of lipid A. The hydrophobic leucine residues and tail of colistin A bind with the fatty acid tails of lipid A, allowing the uptake of colistin A, and disrupt, the bacterial OM. **b (right)**, a model of colistin binding to phosphoethanolamine-1΄-lipid A indicates the addition of positively charged phosphoethanolamine onto the 1′-PO_4_ of lipid A likely interferes with the interaction of positively charged Dab8 and Dab9 side chains with the phosphate group, preventing colistin binding to the outer membrane of GNB. The model B is adapted from Yang *et al*. [74].

MCR-1 is a phosphoethanolamine lipid A transferase enzyme, belonging to the “YhjW/YjdB/YijP” alkaline phosphatase superfamily [73]. The mechanism by which *mcr-1* can mediate colistin resistance does not differ from that found in intrinsically resistant GNB. MCR-1 encodes a PEtN transferase leading to the addition of a PEtN moiety to the lipid A of LPS, increasing the cationic charges on LPS, and consequently, decreases the binding of colistin to LPS [2,16,35,58]. This action is attributed to the chemical structure of the PEtN transferase. The N-terminal region of PEtN transferase is inserted in the inner membrane, while the C-terminal catalytic sulfatase domain is found periplasmic. The latter process is responsible for the transfer of a pEtN moiety from its physiological donor phosphatidylethanolamine to the Kdo of LPS [3,23]. Structure-guided functional studies have confirmed this mechanism of *mcr-1* and revealed that the enzymatic activity of *mcr-1* renders the recipient strains resistant to polymyxin [43,73] (Figure 4).

A previous study reported that the *mcr-1* gene leads to 4- to 8-fold increase in the MICs of colistin in *E. coli*, which indicates that the *mcr-1* alone without other resistance mechanisms is enough to provide resistance against colistin in *E. coli* and other Enterobacteriaceae [23].

An *in silico* analysis of the amino acid sequence of the *mcr-1* gene showed that it is closely related to the PEtN transferases (*pmrC*) found in *Paenibacillus* spp., as well as to other enzymes from GNB, some of which are intrinsically resistant to colistin [23,35,58].

Currently, 22 functional genetic variants of *mcr-1* have been assigned [75–82], including *mcr-1.1* [35], *mcr-1.2* [83], *mcr-1.3* [84], *mcr-1.4* [85], *mcr-1.5* [86], *mcr-1.6* [87], *mcr-1.7* [85], *mcr-1.8* [43], *mcr-1.9* [88], *mcr-1.10* [75], *mcr-1.11* [89], *mcr-1.12* [43], *mcr-1.13* [90], *mcr-1.14* [71], and *mcr-1.15* [91], while the other genetic variants from *mcr-1.16* to *mcr-1.22*, were uploaded to NCBI GenBank (https://www.ncbi.nlm.nih.gov/nuccore/NG_065944.1). These variants differ from *mcr-1* by one or a few amino acids. Therefore, they all share high nucleotide and amino acid identity (∼99%), and thus confer a similar effect on colistin resistance [58,85].

Intriguingly, the determinants of transferable colistin resistance have extended further away *mcr-1* to include a number of novel *mcr-1* alleles [92]. Up to now, nine *mcr* alleles have been reported including *mcr-1* [75–79,81,82,93], namely; *mcr-2* (1617 bp) [79], *mcr-3* (1626 bp) [82], *mcr-4* (1626 bp) [77], *mcr-5* (1644 bp) [76], *mcr-6* (1617 bp) [75], *mcr-7* (1620 bp) [81], *mcr-8* (1698 bp) [78], and the most recently detected *mcr-9* (2661 bp) [93] (Table 2).

**Table 2.** Main characteristics of mcr genes related to polymyxin resistance**.**

Gene	No. of alleles	Associated plasmids and other mobile elements	Coexistence of other resistance genes	Host bacterial species	Potential origin of *mcr* genes	Amino acid identity to *mcr-1*	References
***mcr-1***	22	IncI2, IncX4, IncHI2/HI2A, IncHI1 IncF, IncN, IncP, IncQ, IncX, IncY, IncPO111 Mainly associated with IS*Apl1,* Tn*6330* transposon IS*26*-like element Occasionally chromosomal	*bla*_CTX-M-55/14/15/65/1/2/8/9/27_, *bla*_NDM-1/5/9_ *ampC*, *bla*_IMP-8_, *bla*_SHV12/110_, *bla*_KPC-2_, *bla*_TEM-1/1B/52/135/195_, *bla*_CMY-2_, *bla*_OXA-1/48_, *bla*_VIM-1_ *aph(3’)-la/lb/lv, aac(3)-lva, aph(3'’)-lb, aph(4)-la, aac(6’)lb-cr, aph(6)- ld, aac(6'’)-lb-cr, aadA1/A2, strA, strB sul1, sul2, sul3, tet*(A), *tet*(B) *fosA3, PER, qnrB, qnrS, floR catA, cmlA, dfrA1, dfrA12, qoxAB, arr-3* *mcr-3*, *mcr-4*, *mcr-5*	*E. coli K. pneumoniae Salmonella* spp. *Enterobacter* spp. *Shigella* spp. *Citrobacter* spp. *Moraxella* spp. *K. ascorbata Providencia alcalifaciens R. ornithinolytica C. sakazakii*	*Moraxella porci*	98.7%	[3,5,35,55,58,69,73,75,80,83–90]
***mcr-2***	3	IncX4 May be associated with IS*1595*-like element	Not mentioned	*E. coli Salmonella* spp.	*Moraxella pleuranimalium*	99%	[75,79]
***mcr-3***	30	IncHI2, IncP Tn*As2* transposon	*bla*_CTX-M-55_, *bla*_KPC-2_, *bla*_TEM-1B_ *aac(3)-lld, aac(3)-lva, aph(3’)-la, ant(3'’)-la, aac(6’)-laa, aac(6’)-lb, aac(6’)lb-cr, aph(4)-la, aadA1b, aadA1, aadA2, aadA3, aadA8b, strA, strB sul1, sul2, sul3, tet*(A), *tet*(B) *qnrS1, floR, catA2, cmlA1, catB1, arr-3, dfrA12, dfrA5 mcr-1*, *mcr-9*	*E. coli K. pneumoniae S. enterica Shigella* spp. *Proteus mirabilis Aeromonas* spp.	*Aeromonas* spp.	76–85%	[43,58,93,94]
***mcr-4***	6	ColE IS*Aba19*, IS*5*-like element (IS*Kpn6*), IS*26* Tn*3*-like transposon	*bla*_CTX-M-1/9/14_, *bla*_NDM-1_, *bla*_KPC-2_, *bla*_SHV-12_, *bla*_TEM-1B/135_, *bla*_OXA-67_, *bla*_ADC-6_, *ampC aac(3)-lva, aph(3’)-lc, aph(4)-la, aadA1, aadA2, ant(2'’)-la, strA, strB qnrA, catA1, dfrA1, dfrA16, floR, mph(B) sul1, sul2, tet*(A), *tet*(B) *mcr-5*	*E. coli Shewanella frigidimarina Enterobacter cloacae Salmonella* spp. *Acinetobacter* spp.	*Shewanella* spp.	82–99%	[58,77,91]
***mcr-5***	4	ColE, IncX1 Tn*3-*like transposon(Tn*6542*) Chromosomal	*bla*_TEM-1B_, *bla*_TEM-176_ *aadA1, aadA2, aadA4, aph(3’)-la, aph(4)-la, aph(6)-ld, aac(3)-lva sul1, sul2, sul3, tet*(A), *tet*(B), *tet*(D) *qnrS1, cmlA1-like, mef(B) dfrA1-like, dfrA5, dfrA12 mcr-4*	*S. enterica E. coli P. aeruginosa Aeromonas hydrophila Cupriavidus gilardii*	Maybe from *Cupriavidus gilardii*	36%	[58,76]
***mcr-6***	1	No intact insertion elements	Unclear	*Moraxella* spp.	*Moraxella pleuranimalium*	88% (vs. *mcr-2*)	[36,75]
***mcr-7***	1	IncI2	* bla*_CTX-M-55_	*K. pneumoniae*	*Aeromonas* spp.	69%–81%	[81]
***mcr-8***	4	IncFII IS*903B* IS*Ecl1*	*bla*_NDM_, *bla*_TEM-1B_, *bla*_OXA-1_, *bla*_SHV-73_ *aac(3)-lva, aph(3’)-la, aph(4)-la, aac(6’)-lb, aadA1, aadA2, strA, strB* *qnrS4, oqxAB, qnrB52, qnrB4, sul* genestet genes, *mph(A), mph(E), cat armA, fosA, mph(E), floR, cml*	*K. pneumoniae Raoultella* spp. *Stenotrophomonas* spp.	-	31%	[78,91]
***mcr-9***	2	IncHI2/HI2A, IncFII(s), TrfA Always associated with IS*903B* IS*15DII*, IS*1R*, or IS*26*-like	*bla*_NDM-1_, *bla*_VIM-4_, *bla*_SHV-12_, *bla*_TEM-1/1B_ *aac(6’)-laa, aac(3)-lib, aac(6’)-lic, aph(3’)-la, aph(6)-ld, ant(3'’)-la, aadA2, strA sul1, sul2, tet*(A), *tet*(D) *ere(A), dfrA18, qnrB2, floR mcr-3.17*	*Salmonella* spp. *Klebsiella* spp. *Enterobacter* spp. *Salmonella* spp. *Leclercia* spp. *Citrobacter* spp. *Raoultella* spp. *Phytobacter ursingii C. sakazakii*	*Buttiauxella gaviniae*	84%	[93]

Some of the given data were obtained from PubMed-NCBI GenBank.

Although, all these alleles have been characterized as PEtN transferases, sharing conserved amino acid groups; but, the degree of similarity in amino acid sequences between them is variable, thus reflecting different genetic origins [76]. Investigations on the genetic environment of *mcr* genes revealed that *mcr-2*, *mcr-3*, *mcr-4*, *mcr-5*, *mcr-6*, *mcr-7*, and *mcr-8* share only 81%, 34%, 33%, 31%, 82%, 29%, and 31% amino acid sequence identity with *mcr-1*, respectively [95].

Regarding the recently identified *mcr-9* gene, the three-dimensional (3D) structural models related to all the nine *mcr* homologues (*mcr-1* to *−9*) showed that *mcr-3*, *mcr-4*, *mcr-7*, and *mcr-9*, are sharing a high degree of similarity at the structural level [93].

Indeed, the *mcr-1* gene is the most prevalent among Enterobacteriaceae isolated from human samples [95]. The analysis of the protein structure of MCR-1 showed the presence of two PEtN transferases, namely; LptA and EptC (or *cptA*) from *Neisseria meningitidis* and *Campylobacter jejuni*, respectively, both are intrinsically resistant to polymyxins [35,79]. Of note, *mcr-2* and *mcr-5* are viewed as two infrequent members of the MCR-like protein family [92].

**The *mcr-2*** was subsequently detected in *E. coli* recovered from cattle, and porcine from Belgium [79] and has recently been detected in human vaginal swabs from China [96]. The PEtN transferases encoded by the genes, *mcr-1* (541 aa) and *mcr-2* (538 aa), respectively, share 81% amino acid identity. Their phylogenetic analysis has revealed that they are identical to *Paenibacillus sophorae* and *Moraxella osloensis*, by 63% and 64%, respectively [79].

Three genetic variants of *mcr-2* have been identified until now, namely; *mcr-2.1* [79], *mcr-2.2* [75], and *mcr-2.3* (https://www.ncbi.nlm.nih.gov/nuccore/NG_065452.1).

**The *mcr-3*** was first identified in a swine isolate of *E. coli* in Shandong Province, China [82]. Phylogenetic analysis has indicated that *mcr-3* is evolutionarily distinct from *mcr-1* and closely related to PEtN from *Aeromonas* spp. [58]. *mcr-3* seems to be second only to *mcr-1*; it has been identified in Asia [97], Europe [94], and North America [98].

Sequence alignment proposed that *mcr-3* (541 aa) has a notable high similarity to the chromosome-encoded EptA (53.1%) than to *mcr-1* (44.1%). This is harmonious with the fact that *mcr-3* is clustered in a subclade distinct from that of *mcr-1* (or *mcr-2*). Moreover, it has been reported that *mcr-3* is a comparatively weak version of MCR-like enzymes since the MIC of colistin is 2 µg/ml for *mcr-3*, while 4 µg/ml for *mcr-1*, thus the coexistence of *mcr-1* and *mcr-3* does not confer a significant additive influence on polymyxin resistance [43].

Up to now, 30 functional genetic variants of *mcr-3* have been identified, from *mcr-3.1* to *mcr-3.30* (https://www.ncbi.nlm.nih.gov/nuccore/NG_065456.1.).

**The *mcr-4*** has been identified first in *S. enterica* isolated from a pig on an 8,749 bp ColE10 plasmid in Italy [77,92]. Sequence analysis showed that *mcr-4* has 34.0%, 35.0%, and 49.0% amino acid sequence identity to *mcr-1*, *mcr-2*, and *mcr-3*, respectively. It has been suggested that *mcr-4* may have emerged from a *Shewanella* species (a bacterium frequently presents in aquatic niches) [58]. Currently, six variants of *mcr-4* have been identified, including *mcr-4.1* to *mcr-4.6*.

**The *mcr-5*** has been detected in *S. Paratyphi B* dTa+ from poultry in Germany [76]. Its protein analysis revealed sequence homology with MCR-1, MCR-2, MCR-3, and MCR-4 by 36.11%, 35.29%, 34.72%, and 33.71%, respectively. Sequence analysis proposed that *mcr-5* may have emerged from the environmental *Cupriavidus gilardii* [58]. Up to now, four genetic variants of *mcr-5* are identified, namely; *mcr-5.1* to *mcr-5.4* (https://www.ncbi.nlm.nih.gov/nuccore/NG_065945.1).

**The *mcr-6*** (previously known as *mcr-2.2*) has been discovered in *Moraxella* spp. isolated from pigs in Great Britain. Besides, only one variant of *mcr-6* (*mcr-6.1*) has been identified until now [36].

**The *mcr-7*** has been identified in *K. pneumoniae* isolated from chickens in China and also one variant of *mcr-7* (*mcr-7.1*) has been identified until now [81].

**The *mcr-8*** was found in NDM-producing *K. pneumoniae* isolated from both pigs and humans in China [78]. Four variants of *mcr-8* were identified, including; *mcr-8.1* to *mcr-8.4* [91].

Eventually, Carroll *et al*. [93] have identified the novel *mcr* homologue, *mcr-9*, which was isolated from *S. Typhimurium* strain HUM_TYPH_WA_10_R9_3274. Investigations on the genetic environment of *mcr-9* gene revealed that the amino acid sequence of *mcr-9* most closely resembled those of *mcr-3* and *mcr-7*. Mcr-3.17 has the highest-scoring *mcr* allele, which shares 64.5% amino acid sequence identity with *mcr-9* and 99.5% coverage [93].

Besides, two variants of *mcr-9* have been identified until now, namely; *mcr-9.1* and *mcr-9.2* (https://www.ncbi.nlm.nih.gov/nuccore/1704734405).

## Diversity in *mcr*-harbouring plasmid reservoirs

A serious concern regarding *mcr* genes is their location on transferable plasmids. The *mcr-1* gene was identified for the first time in an IncI2 plasmid named pHNSHP45 (64 105 bp) [3,36]. Following this initial plasmid detection, several *mcr-1*-carrying plasmids have been reported belonging to different incompatibility groups with various sizes (58–251 kb) [23]; IncI2 [35], IncHI2 [72], IncX4 [73], IncP [55], IncY, IncF, IncFI, IncFII, IncFIB, IncK2 [43], IncN, and IncQ [36] plasmids (Table 2).

Moreover, many other replicon types of plasmids were specified to harbour *mcr*-like genes, indicating that the *mcr-1*-like variants might have been circulated worldwide by multiple plasmids. As a result of the low global spreading rate of the other *mcr*-like variants, the replicon types of plasmids harbouring these *mcr*-like variants are very scarce [43].

Intriguingly, it has been demonstrated that two different *mcr-1*-harbouring plasmids can coexist in a single colistin-resistant *E. coli* isolate, such as the IncI2-type plasmid, pGD65-3, and the IncX4-like plasmid, pGD65-4 [70]. The *mcr-2* gene is only found in an IncX4 plasmid named pKP37-BE (35 104 bp) [79], whereas the *mcr-3* gene has been identified in both the IncHI2 plasmid [82,94] and the IncP plasmid [97]. Besides, *mcr-4* and *mcr-5* genes have been shown able to transfer by the same ColE-type plasmid with relatively-small size [76,77].

Sequence analysis of *mcr* genes showed that the *mcr-1* gene is often accompanied by an ISApl1 insertion sequence (IS), which is located upstream [5,35]. It has been reported that the ISApl1, which is located downstream of *mcr-1* is not as stable as it does in the upstream of *mcr-1* [43]. Generally, ISApl1 is flanked with *mcr-1* and contributes to its transposition [3,5]. Besides, another IS, namely IS1 might also appear upstream of the *mcr-1* gene [43].

Analysing the genetic environment of *mcr-2* revealed similarity with that of *mcr-1*, where an IS belonging to the IS1595 superfamily is found upstream of *mcr-2* [3,43]. Regarding the *mcr-3* gene, the transposon TnAs2 occurs upstream of nimC/nimA-*mcr-3* [82,97], whereas, in the *mcr-4*-positive ColE10-type plasmid, the ISKpn6 (IS5 element) is located upstream of *mcr-4*. Besides, the *mcr-5* gene has been found within a Tn3-family transposon carried on a 12-kb ColE-type plasmid [76].

Intriguingly, Poirel *et al*. [23] revealed that the *mcr-1* gene is located within a 2,600-bp genetic structure, called the “*mcr-1* cassette,” that might have been mobilized by transposition. The cassette was noticed carrying its promoter sequences leading to the *mcr-1* expression.

The *mcr* genes have altered the scenario of colistin resistance since they have become a probable menace to public health. Furthermore, some, but not all plasmids-harbouring the *mcr-1* gene can encode other antibiotic resistance genes, such as *bla*_CTX-M_, *floR* and/or *qnr*, which can encode resistance to various antibiotic classes, including polymyxins, β-lactams, quinolones [94], tetracyclines [23], and amphenicols [36].

Of note, the position of the *mcr-1* gene on MDR-plasmids is worrisome, since upon the use of antibiotics other than polymyxins this will lead to co-selection for the isolates that harbour *mcr-1* and facilitate its dissemination [23]. More worryingly, is the integration of the *mcr-1* gene into the bacterial chromosome, which has been discovered to occur in some strains [3]. For instance, in Switzerland, the integration of the *mcr-1* gene has been detected on the chromosome of an *E. coli* strain, which indicates that the *mcr-1* gene might be integrated and consequently, stabilized in the genome of some isolates [23].

The higher occurrence of the *mcr-1* gene in bacteria carrying genes coding for carbapenemases and/or ESBLs (e.g. CTX-M-15 and CTX-M-55) is most probably due to various and complex genetic events selected under antibiotic pressure [3] (Table 2). For instance, in a previous study, the co-transfer of *mcr-1* and *bla*_CTX-M-1_ genes, which are located on the IncHI2 plasmid of *S. enterica* isolated from retail swine meat by horizontal gene transfer under colistin selection has been reported. Indeed, this hinders the therapeutic options for the treatment of *S. enterica* infections. [36].

Besides, the existence of the *mcr-1* gene has been reported in high drug-resistant Enterobacteriaceae isolates harbouring plasmids encoding different carbapenemase genes (*bla*_NDM-1_, *bla*_NDM-5_, *bla*_NDM-9_, *bla*_OXA-48_, *bla*_KPC-2_, and *bla*_VIM-1_). For instance, the simultaneous presence of *mcr-1* and the Metallo-β-lactamase NDM-5 has been detected in *K. pneumoniae* clinical isolate [3,72]. These findings reflect the possibility of emerging a severe public health crisis due to Enterobacteriaceae isolates harbouring both *mcr-1* and carbapenemase-encoding genes.

## Future prospects for polymyxins resistance

Investigations in polymyxins resistance have led to the detection of different mechanisms of resistance contributing to their resistance. Additionally, new mechanisms were discovered in resistant strains with previously unexplained mechanism(s). However, there are still lots of unknowns relating to polymyxin resistance. For instance, there still exist some resistant bacterial strains with an unknown mechanism that requires further investigations.

Besides, some bacterial species are possessing intrinsic colistin resistance. Such resistance has been attributed to the presence of LPSs being modified with L-Ara4N, explaining their intrinsic resistance [99]. Xu *et al*. [100] reported that the naturally occurring colistin resistance is attributed to the functional expression of specific chromosomal genes such as *eptA* of *N. meningitidis*. Deciphering other reason(s) behind such resistance will clarify certain mechanisms of polymyxin resistance that are still unclear.

Eventually, as is already known, colistin resistance mostly follows the exposure to colistin. However, it has been revealed that the colistin resistance can be developed without any prior colistin exposure. This represents a severe menace, which hinders the use of colistin as a last resort against MDR GNB. An understanding of this phenomena is crucial to guard against the future possibility of the development of PDR strains encoding colistin resistance.

## Conclusions

Polymyxins have been used for several decades as bactericidal agents against intractable GNB. As a result of their adverse toxic effects, their use has been limited or even stopped. However, they have been reintroduced in clinical practice as a last resort against MDR GNB. They act by disrupting the bacterial outer and inner membranes, resulting in cellular death. The primary mechanism of resistance is via the modification of the bacterial OM, which is mostly attributed to the PmrA-PmrB and PhoP-PhoQ TCSs. Additionally, heteroresistance to polymyxins is an emerging menace attributed to the bacterial exposure to suboptimal polymyxin dosages and represents a potential source of colistin resistance.

The emergence of the plasmid-mediated *mcr-1* gene encoding for colistin resistance in GNB, which is transferable between different bacterial species has highlighted the possibility of losing colistin efficiency against MDR GNB in humans. Up to now, 22 new genetic variants of *mcr-1* have been identified in different countries, indicating the possibility of continuous evolution. Besides, a number of novel *mcr*-1 alleles have been reported including *mcr-1*, namely; *mcr-2*, *mcr-3*, *mcr-4*, *mcr-5*, *mcr-6*, *mcr-7*, *mcr-8*, and the very most recently detected *mcr-9*.

Therefore, prospective surveillance and epidemiological studies should be implemented to detect the rate of dissemination of this resistant-gene in humans as well as in animals.

Herein, we aimed to provide an overview of all possible mechanisms of polymyxins resistance described till now. Indeed, there are still many unknown mechanisms of resistance that require more investigations to detect their exact role, which in turn, will improve our understanding about how to overcome polymyxins resistance and will permit the potentiality to develop more potent and less toxic polymyxin derivatives.

## References

[1] Falagas ME, Kasiakou SK. Colistin: the revival of polymyxins for the management of multidrug-resistant gram-negative bacterial infections. Clin Infect Dis. 2005;40(9):1333–1341. 15825037 10.1086/429323

[2] Sun J, Zhang H, Liu YH, et al. Towards understanding MCR-like colistin resistance. Trends Microbiol. 2018;26(9):794–808. 29525421 10.1016/j.tim.2018.02.006

[3] Jeannot K, Bolard A, Plesiat P. Resistance to polymyxins in Gram-negative organisms. Int J Antimicrob Agents. 2017;49(5):526–535. 28163137 10.1016/j.ijantimicag.2016.11.029

[4] Srinivas P, Rivard K. Polymyxin resistance in Gram-negative pathogens. Curr Infect Dis Rep. 2017;19(11):38. 28895051 10.1007/s11908-017-0596-3

[5] Sherry N, Howden B. Emerging Gram negative resistance to last-line antimicrobial agents fosfomycin, colistin and ceftazidime-avibactam - epidemiology, laboratory detection and treatment implications. Expert Rev Anti Infect Ther. 2018;16(4):289–306. 29543500 10.1080/14787210.2018.1453807

[6] Velkov T, Roberts KD, Nation RL, et al. Pharmacology of polymyxins: new insights into an ‘old’ class of antibiotics. Future Microbiol. 2013;8(6):711–724. 23701329 10.2217/fmb.13.39 PMC3852176

[7] Talbot GH, Bradley J, Edwards JE, Jr., et al. Bad bugs need drugs: an update on the development pipeline from the antimicrobial availability task force of the infectious diseases society of America. Clin Infect Dis. 2006;42(5):657–668. 16447111 10.1086/499819

[8] Bialvaei AZ, Samadi KH. Colistin, mechanisms and prevalence of resistance. Curr Med Res Opin. 2015;31(4):707–721. 25697677 10.1185/03007995.2015.1018989

[9] Lim LM, Ly N, Anderson D, et al. Resurgence of colistin: a review of resistance, toxicity, pharmacodynamics, and dosing. Pharmacotherapy. 2010;30(12):1279–1291. 21114395 10.1592/phco.30.12.1279 PMC4410713

[10] Dijkmans AC, Wilms EB, Kamerling IM, et al. Colistin: revival of an old polymyxin antibiotic. Ther Drug Monit. 2015;37(4):419–427. 25549206 10.1097/FTD.0000000000000172

[11] Son SJ, Huang R, Squire CJ, et al. MCR-1: a promising target for structure-based design of inhibitors to tackle polymyxin resistance. Drug Discov Today. 2019;24(1):206–216. 30036574 10.1016/j.drudis.2018.07.004

[12] Koyama Y. A new antibiotic “colistin” produced by spore-forming soil bacteria. J Antibiot. 1950 1950;3:457–458.

[13] Kaye KS, Pogue JM, Tran TB, et al. Agents of last resort: polymyxin resistance. Infect Dis Clin North Am. 2016;30(2):391–414. 27208765 10.1016/j.idc.2016.02.005

[14] Falagas ME, Rafailidis PI, Matthaiou DK. Resistance to polymyxins: mechanisms, frequency and treatment options. Drug Resist Updat. 2010;13(4–5):132–138. 20843473 10.1016/j.drup.2010.05.002

[15] Gurjar M. Colistin for lung infection: an update. J Intensive Care. 2015;3(1):3. 25705428 10.1186/s40560-015-0072-9 PMC4336271

[16] Li J, Nation RL, Milne RW, et al. Evaluation of colistin as an agent against multi-resistant Gram-negative bacteria. Int J Antimicrob Agents. 2005;25(1):11–25. 15620821 10.1016/j.ijantimicag.2004.10.001

[17] Antonic V, Stojadinovic A, Zhang B, et al. *Pseudomonas aeruginosa* induces pigment production and enhances virulence in a white phenotypic variant of *Staphylococcus aureus*. Infect Drug Resist. 2013;6:175–186. 24232573 10.2147/IDR.S49039 PMC3825675

[18] Mlynarcik P, Kolar M. Molecular mechanisms of polymyxin resistance and detection of *mcr* genes. Biomed Pap Med Fac Univ Palacky Olomouc Czech Repub. 2019;163(1):28–38. 30439931 10.5507/bp.2018.070

[19] Abachin E, Poyart C, Pellegrini E, et al. Formation of D-alanyl-lipoteichoic acid is required for adhesion and virulence of *Listeria monocytogenes*. Mol Microbiol. 2002;43(1):1–14. 11849532 10.1046/j.1365-2958.2002.02723.x

[20] Gregoire N, Aranzana-Climent V, Magreault S, et al. Clinical Pharmacokinetics and Pharmacodynamics of colistin. Clin Pharmacokinet. 2017;56(12):1441–1460. 28550595 10.1007/s40262-017-0561-1

[21] Liberati A, Altman DG, Tetzlaff J, et al. The PRISMA statement for reporting systematic reviews and meta-analyses of studies that evaluate health care interventions: explanation and elaboration. PLoS Med. 2009;6(7):e1000100. 19621070 10.1371/journal.pmed.1000100 PMC2707010

[22] Rhouma M, Beaudry F, Theriault W, et al. Colistin in Pig production: chemistry, mechanism of antibacterial action, microbial resistance emergence, and one health perspectives. Front Microbiol. 2016;7:1789. 27891118 10.3389/fmicb.2016.01789 PMC5104958

[23] Poirel L, Jayol A, Nordmann P. Polymyxins: antibacterial activity, susceptibility testing, and resistance mechanisms encoded by plasmids or chromosomes. Clin Microbiol Rev. 2017;30(2):557–596. 28275006 10.1128/CMR.00064-16 PMC5355641

[24] Yu Z, Qin W, Lin J, et al. Antibacterial mechanisms of polymyxin and bacterial resistance. Biomed Res Int. 2015;2015:679109. 10.1155/2015/679109 PMC4312571 25664322

[25] Yu Z, Zhu Y, Fu J, et al. Enhanced NADH metabolism involves colistin-induced killing of *Bacillus subtilis* and *Paenibacillus polymyxa*. Molecules. 2019;24(3):387. 30678237 10.3390/molecules24030387 PMC6384706

[26] Mogi T, Murase Y, Mori M, et al. Polymyxin B identified as an inhibitor of alternative NADH dehydrogenase and malate: quinone oxidoreductase from the Gram-positive bacterium *Mycobacterium smegmatis*. J Biochem. 2009;146(4):491–499. 19564154 10.1093/jb/mvp096

[27] Deris ZZ, Akter J, Sivanesan S, et al. A secondary mode of action of polymyxins against Gram-negative bacteria involves the inhibition of NADH-quinone oxidoreductase activity. J Antibiot. 2014;67(2):147–151. 10.1038/ja.2013.111 PMC3943757 24169795

[28] Martis N, Leroy S, Blanc V. Colistin in multi-drug resistant *Pseudomonas aeruginosa* blood-stream infections: a narrative review for the clinician. J Infect. 2014;69(1):1–12. 24631777 10.1016/j.jinf.2014.03.001

[29] Grau-Campistany A, Manresa A, Pujol M, et al. Tryptophan-containing lipopeptide antibiotics derived from polymyxin B with activity against Gram positive and Gram negative bacteria. Biochim Biophys Acta. 2016;1858(2):333–343. 26607008 10.1016/j.bbamem.2015.11.011

[30] Domalaon R, Berry L, Tays Q, et al. Development of dilipid polymyxins: Investigation on the effect of hydrophobicity through its fatty acyl component. Bioorg Chem. 2018;80:639–648. 30053708 10.1016/j.bioorg.2018.07.018

[31] Rose F, Heuer KU, Sibelius U, et al. Targeting lipopolysaccharides by the nontoxic polymyxin B nonapeptide sensitizes resistant *Escherichia coli* to the bactericidal effect of human neutrophils. J Infect Dis. 2000;182(1):191–199. 10882597 10.1086/315669

[32] Vaara M. Polymyxin derivatives that sensitize Gram-negative bacteria to other antibiotics. Molecules. 2019;24(2):249. 30641878 10.3390/molecules24020249 PMC6359160

[33] MacNair CR, Stokes JM, Carfrae LA, et al. Overcoming *mcr-1* mediated colistin resistance with colistin in combination with other antibiotics. Nat Commun. 2018;9(1):458. 29386620 10.1038/s41467-018-02875-z PMC5792607

[34] Hood MI, Becker KW, Roux CM, et al. . genetic determinants of intrinsic colistin tolerance in *Acinetobacter baumannii*. Infect Immun. 2013;81(2):542–551. 23230287 10.1128/IAI.00704-12 PMC3553813

[35] Liu YY, Wang Y, Walsh TR, et al. Emergence of plasmid-mediated colistin resistance mechanism MCR-1 in animals and human beings in China: a microbiological and molecular biological study. Lancet Infect Dis. 2016;16(2):161–168. 26603172 10.1016/S1473-3099(15)00424-7

[36] Lima T, Domingues S, Da Silva GJ. Plasmid-mediated colistin resistance in *Salmonella enterica*: A review. Microorganisms. 2019;7(2):55. 30791454 10.3390/microorganisms7020055 PMC6406434

[37] Cannatelli A, Giani T, D'Andrea MM, et al. Mgrb inactivation is a common mechanism of colistin resistance in KPC-producing *Klebsiella pneumoniae* of clinical origin. Antimicrob Agents Chemother. 2014;58(10):5696–5703. 25022583 10.1128/AAC.03110-14 PMC4187966

[38] Lippa AM, Goulian M. Feedback inhibition in the PhoQ/PhoP signaling system by a membrane peptide. PLoS Genet. 2009;5(12):e1000788. 20041203 10.1371/journal.pgen.1000788 PMC2789325

[39] Cheng YH, Lin TL, Lin YT, et al. Amino acid substitutions of CrrB responsible for resistance to colistin through CrrC in *Klebsiella pneumoniae*. Antimicrob Agents Chemother. 2016;60(6):3709–3716. 27067316 10.1128/AAC.00009-16 PMC4879426

[40] Perez F, El Chakhtoura NG, Yasmin M, et al. Polymyxins: To Combine or Not to Combine? Antibiotics. 2019;8(2):38. 30974813 10.3390/antibiotics8020038 PMC6627991

[41] Fresno S, Jimenez N, Izquierdo L, et al. The ionic interaction of *Klebsiella pneumoniae* K2 capsule and core lipopolysaccharide. Microbiology. 2006;152(Pt 6):1807–1818. 16735743 10.1099/mic.0.28611-0

[42] Lima WG, Alves MC, Cruz WS, et al. Chromosomally encoded and plasmid-mediated polymyxins resistance in *Acinetobacter baumannii*: a huge public health threat. Eur J Clin Microbiol Infect Dis. 2018;37(6):1009–1019. 29524060 10.1007/s10096-018-3223-9

[43] Feng Y. Transferability of MCR-1/2 polymyxin resistance: complex dissemination and genetic mechanism. ACS Infect Dis. 2018;4(3):291–300. 29397687 10.1021/acsinfecdis.7b00201

[44] Snitkin ES, Zelazny AM, Gupta J, et al. Genomic insights into the fate of colistin resistance and *Acinetobacter baumannii* during patient treatment. Genome Res. 2013;23(7):1155–1162. 23564252 10.1101/gr.154328.112 PMC3698508

[45] Adams MD, Nickel GC, Bajaksouzian S, et al. Resistance to colistin in *Acinetobacter baumannii* associated with mutations in the PmrAB two-component system. Antimicrob Agents Chemother. 2009;53(9):3628–3634. 19528270 10.1128/AAC.00284-09 PMC2737849

[46] Cheah SE, Johnson MD, Zhu Y, et al. Polymyxin resistance in *Acinetobacter baumannii*: genetic mutations and transcriptomic changes in response to clinically relevant dosage regimens. Sci Rep. 2016;6:26233. 27195897 10.1038/srep26233 PMC4872528

[47] Moffatt JH, Harper M, Harrison P, et al. Colistin resistance in *Acinetobacter baumannii* is mediated by complete loss of lipopolysaccharide production. Antimicrob Agents Chemother. 2010;54(12):4971–4977. 20855724 10.1128/AAC.00834-10 PMC2981238

[48] Bojkovic J, Richie DL, Six DA, et al. Characterization of an *Acinetobacter baumannii lptD* deletion strain: permeability defects and response to inhibition of lipopolysaccharide and fatty acid biosynthesis. J Bacteriol. 2015;198(4):731–741. 26668262 10.1128/JB.00639-15 PMC4751815

[49] Whitfield C, Trent MS. Biosynthesis and export of bacterial lipopolysaccharides. Annu Rev Biochem. 2014;83:99–128. 24580642 10.1146/annurev-biochem-060713-035600

[50] Thi Khanh NN, Riordan DW, Do Hoang NT, et al. The induction and identification of novel colistin resistance mutations in *Acinetobacter baumannii* and their implications. Sci Rep. 2016;6:28291. 27329501 10.1038/srep28291 PMC4916428

[51] Lin MF, Lin YY, Lan CY. Contribution of EmrAB efflux pumps to colistin resistance in *Acinetobacter baumannii*. J Microbiol. 2017;55(2):130–136. 28120193 10.1007/s12275-017-6408-5

[52] Trebosc V, Gartenmann S, Totzl M, et al. Dissecting colistin resistance mechanisms in extensively drug-resistant *Acinetobacter baumannii* clinical isolates. MBio. 2019;10(4):e01083-19. 31311879 10.1128/mBio.01083-19 PMC6635527

[53] Muller C, Plesiat P, Jeannot K. A two-component regulatory system interconnects resistance to polymyxins, aminoglycosides, fluoroquinolones, and beta-lactams in Pseudomonas aeruginosa. Antimicrob Agents Chemother. 2011;55(3):1211–1221. 21149619 10.1128/AAC.01252-10 PMC3067119

[54] Miller AK, Brannon MK, Stevens L, et al. Phoq mutations promote lipid A modification and polymyxin resistance of *Pseudomonas aeruginosa* found in colistin-treated cystic fibrosis patients. Antimicrob Agents Chemother. 2011;55(12):5761–5769. 21968359 10.1128/AAC.05391-11 PMC3232818

[55] Zhao F, Feng Y, Lu X, et al. Incp plasmid carrying colistin resistance gene *mcr-1* in *Klebsiella pneumoniae* from hospital sewage. Antimicrob Agents Chemother. 2017;61(2):e02229-16. 27895009 10.1128/AAC.02229-16 PMC5278755

[56] Lee JY, Na IY, Park YK, et al. Genomic variations between colistin-susceptible and -resistant *Pseudomonas aeruginosa* clinical isolates and their effects on colistin resistance. J Antimicrob Chemother. 2014;69(5):1248–1256. 24474431 10.1093/jac/dkt531

[57] Morey P, Viadas C, Euba B, et al. Relative contributions of lipooligosaccharide inner and outer core modifications to nontypeable *Haemophilus influenzae* pathogenesis. Infect Immun. 2013;81(11):4100–4111. 23980106 10.1128/IAI.00492-13 PMC3811809

[58] Quiroga C, Nastro M, Di CJ. Current scenario of plasmid-mediated colistin resistance in Latin America. Rev Argent Microbiol. 2019;51(1):93–100. 29945744 10.1016/j.ram.2018.05.001

[59] Nikaido H. Molecular basis of bacterial outer membrane permeability revisited. Microbiol Mol Biol Rev. 2003;67(4):593–656. 14665678 10.1128/MMBR.67.4.593-656.2003 PMC309051

[60] Olaitan AO, Morand S, Rolain JM. Mechanisms of polymyxin resistance: acquired and intrinsic resistance in bacteria. Front Microbiol. 2014;5: 643. 25505462 10.3389/fmicb.2014.00643 PMC4244539

[61] Aghapour Z, Gholizadeh P, Ganbarov K, et al. Molecular mechanisms related to colistin resistance in Enterobacteriaceae. Infect Drug Resist. 2019;12:965–975. 31190901 10.2147/IDR.S199844 PMC6519339

[62] Cannatelli A, D'Andrea MM, Giani T, et al. In vivo emergence of colistin resistance in *Klebsiella pneumoniae* producing KPC-type carbapenemases mediated by insertional inactivation of the PhoQ/PhoP *mgrB* regulator. Antimicrob Agents Chemother. 2013;57(11):5521–5526. 23979739 10.1128/AAC.01480-13 PMC3811314

[63] Mills G, Dumigan A, Kidd T, et al. Identification and characterization of two *Klebsiella pneumoniae lpxL* lipid A late Acyltransferases and their role in Virulence. Infect Immun. 2017;85(9):e00068-17. 28652313 10.1128/IAI.00068-17 PMC5563558

[64] Gefen O, Chekol B, Strahilevitz J, et al. TDtest: easy detection of bacterial tolerance and persistence in clinical isolates by a modified disk-diffusion assay. Sci Rep. 2017;7:41284. 28145464 10.1038/srep41284 PMC5286521

[65] Herrera CM, Hankins JV, Trent MS. Activation of PmrA inhibits LpxT-dependent phosphorylation of lipid A promoting resistance to antimicrobial peptides. Mol Microbiol. 2010;76(6):1444–1460. 20384697 10.1111/j.1365-2958.2010.07150.x PMC2904496

[66] Trimble MJ, Mlynarcik P, Kolar M, et al. Polymyxin: alternative mechanisms of action and resistance. Cold Spring Harb Perspect Med. 2016;6(10):a025288. 27503996 10.1101/cshperspect.a025288 PMC5046685

[67] Da Silva GJ, Domingues S. Interplay between colistin resistance, virulence and fitness in *Acinetobacter baumannii*. Antibiotics. 2017;6(4):28. 29160808 10.3390/antibiotics6040028 PMC5745471

[68] Chambers JR, Sauer K. The MerR-like regulator BrlR impairs *Pseudomonas aeruginosa* biofilm tolerance to colistin by repressing PhoPQ. J Bacteriol. 2013;195(20):4678–4688. 23935054 10.1128/JB.00834-13 PMC3807428

[69] Haenni M, Poirel L, Kieffer N, et al. Co-occurrence of extended spectrum beta lactamase and MCR-1 encoding genes on plasmids. Lancet Infect Dis. 2016;16(3):281–282. 10.1016/S1473-3099(16)00007-4 26774244

[70] Wang Q, Sun J, Li J, et al. Expanding landscapes of the diversified *mcr-1*-bearing plasmid reservoirs. Microbiome. 2017;5(1):70. 28683827 10.1186/s40168-017-0288-0 PMC5500976

[71] Wang Y, Hu Y, Cao J, et al. Antibiotic resistance gene reservoir in live poultry markets. J Infect. 2019;78(6):445–453. 30935879 10.1016/j.jinf.2019.03.012

[72] Yang RS, Feng Y, Lv XY, et al. Emergence of NDM-5- and MCR-1-producing *Escherichia coli* Clones ST648 and ST156 from a single Muscovy Duck (Cairina moschata). Antimicrob Agents Chemother. 2016;60(11):6899–6902. 27550364 10.1128/AAC.01365-16 PMC5075103

[73] Gao R, Hu Y, Li Z, et al. Dissemination and mechanism for the MCR-1 colistin resistance. PLoS Pathog. 2016;12(11):e1005957. 27893854 10.1371/journal.ppat.1005957 PMC5125707

[74] Yang Q, Li M, Spiller OB, et al. Balancing *mcr-1* expression and bacterial survival is a delicate equilibrium between essential cellular defence mechanisms. Nat Commun. 2017;8(1):2054. 29233990 10.1038/s41467-017-02149-0 PMC5727292

[75] AbuOun M, Stubberfield EJ, Duggett NA, et al. . *mcr-1* and *mcr-2* variant genes identified in *Moraxella* species isolated from pigs in Great Britain from 2014 to 2015. J Antimicrob Chemother. 2017;72(10):2745–2749. 29091227 10.1093/jac/dkx286 PMC5890717

[76] Borowiak M, Fischer J, Hammerl JA, et al. Identification of a novel transposon-associated phosphoethanolamine transferase gene, *mcr-5*, conferring colistin resistance in d-tartrate fermenting *Salmonella enterica* subsp. *enterica* serovar *Paratyphi B*. J Antimicrob Chemother. 2017;72(12):3317–3324. 28962028 10.1093/jac/dkx327

[77] Carattoli A, Villa L, Feudi C, et al. Novel plasmid-mediated colistin resistance *mcr-4* gene in *Salmonella* and *Escherichia coli*, Italy 2013, Spain and Belgium, 2015 to 2016. Euro Surveill. 2017;22(31):30589. 28797329 10.2807/1560-7917.ES.2017.22.31.30589 PMC5553062

[78] Wang X, Wang Y, Zhou Y, et al. Emergence of a novel mobile colistin resistance gene, *mcr-8*, in NDM-producing *Klebsiella pneumoniae*. Emerg Microbes Infect. 2018;7(1):122. 29970891 10.1038/s41426-018-0124-z PMC6030107

[79] Xavier BB, Lammens C, Ruhal R, et al. Identification of a novel plasmid-mediated colistin-resistance gene, *mcr-2*, in *Escherichia coli*, Belgium, June 2016. Euro Surveill. 2016;21(27):30280. 10.2807/1560-7917.ES.2016.21.27.30280 27416987

[80] Yang F, Shen C, Zheng X, et al. Plasmid-mediated colistin resistance gene *mcr-1* in *Escherichia coli* and *Klebsiella pneumoniae* isolated from market retail fruits in Guangzhou, China. Infect Drug Resist. 2019;12:385–389. 30809099 10.2147/IDR.S194635 PMC6377047

[81] Yang YQ, Li YX, Lei CW, et al. Novel plasmid-mediated colistin resistance gene *mcr-7.1* in *Klebsiella pneumoniae*. J Antimicrob Chemother. 2018;73(7):1791–1795. 29912417 10.1093/jac/dky111

[82] Yin W, Li H, Shen Y, et al. Novel plasmid-mediated colistin resistance gene *mcr-3* in *Escherichia coli*. MBio. 2017;8(3):e00543-17. 28655818 10.1128/mBio.00543-17 PMC5487729

[83] Di P V, Arena F, Tascini C, et al. . *mcr-1.2*, a new *mcr* variant carried on a transferable plasmid from a colistin-resistant KPC carbapenemase-producing *Klebsiella pneumoniae* strain of sequence type 512. Antimicrob Agents Chemother. 2016;60(9):5612–5615. 27401575 10.1128/AAC.01075-16 PMC4997870

[84] Yang YQ, Li YX, Song T, et al. Colistin resistance gene *mcr-1* and its variant in *Escherichia coli* isolates from chickens in China. Antimicrob Agents Chemother. 2017;61(5):e01204-16. 28242671 10.1128/AAC.01204-16 PMC5404584

[85] Zhao F, Feng Y, Lu X, et al. Remarkable diversity of *Escherichia coli* carrying *mcr-1* from hospital sewage with the identification of two New *mcr-1* variants. Front Microbiol. 2017;8:2094. 29118748 10.3389/fmicb.2017.02094 PMC5660977

[86] Tijet N, Faccone D, Rapoport M, et al. Molecular characteristics of *mcr-1*-carrying plasmids and new *mcr-1* variant recovered from polyclonal clinical *Escherichia coli* from Argentina and Canada. PLoS One. 2017;12(7):e0180347. 28678874 10.1371/journal.pone.0180347 PMC5498056

[87] Lu X, Hu Y, Luo M, et al. MCR-1.6, a new MCR variant carried by an IncP plasmid in a colistin-resistant *Salmonella enterica* serovar *Typhimurium* isolate from a healthy individual. Antimicrob Agents Chemother. 2017;61(5):e02632-16. 28264851 10.1128/AAC.02632-16 PMC5404552

[88] Manageiro V, Clemente L, Romao R, et al. Incx4 plasmid carrying the New *mcr-1.9* gene variant in a CTX-M-8-producing *Escherichia coli* isolate recovered from swine. Front Microbiol. 2019;10:367. 30923516 10.3389/fmicb.2019.00367 PMC6426780

[89] Deshpande LM, Hubler C, Davis AP, et al. Updated prevalence of *mcr*-like genes among *Escherichia coli* and *Klebsiella pneumoniae* in the SENTRY program and characterization of *mcr-1.11* variant. Antimicrob Agents Chemother. 2019;63(4):e02450-18. 30917984 10.1128/AAC.02450-18 PMC6437474

[90] Alba P, Leekitcharoenphon P, Franco A, et al. Molecular epidemiology of *mcr*-encoded colistin resistance in enterobacteriaceae from food-producing animals in Italy revealed through the EU harmonized antimicrobial resistance Monitoring. Front Microbiol. 2018;9:1217. 29951045 10.3389/fmicb.2018.01217 PMC6008537

[91] Ma F, Shen C, Zheng X, et al. Identification of a novel plasmid carries *mcr-4.3* in *Acinetobacter baumannii* from China. Antimicrob Agents Chemother. 2019;63(6):e00133-19. 30936095 10.1128/AAC.00133-19 PMC6535571

[92] Xu Y, Zhong LL, Srinivas S, et al. Spread of MCR-3 colistin resistance in China: An epidemiological, Genomic and Mechanistic study. EBioMedicine. 2018;34:139–157. 30061009 10.1016/j.ebiom.2018.07.027 PMC6116419

[93] Carroll LM, Gaballa A, Guldimann C, et al. Identification of novel mobilized colistin resistance gene *mcr-9* in a multidrug-resistant, colistin-susceptible *Salmonella enterica* Serotype *Typhimurium* isolate. MBio. 2019;10(3):e00853-19. 31064835 10.1128/mBio.00853-19 PMC6509194

[94] Litrup E, Kiil K, Hammerum AM, et al. Plasmid-borne colistin resistance gene *mcr-3* in *Salmonella* isolates from human infections, Denmark, 2009–17. Euro Surveill. 2017;22(31):30587. 28797325 10.2807/1560-7917.ES.2017.22.31.30587 PMC5553060

[95] Volland H, Dortet L, Bernabeu S, et al. Development and multicentric validation of a lateral flow immunoassay for rapid detection of MCR-1-producing enterobacteriaceae. J Clin Microbiol. 2019;57(5):e01454-18. 30842227 10.1128/JCM.01454-18 PMC6498016

[96] Zhang J, Chen L, Wang J, et al. Molecular detection of colistin resistance genes (*mcr-1* to *mcr-5*) in human vaginal swabs. BMC Res Notes. 2018;11(1):143. 29463301 10.1186/s13104-018-3255-3 PMC5819219

[97] Liu L, Feng Y, Zhang X, et al. New variant of *mcr-3* in an extensively drug-resistant *Escherichia coli* clinical isolate carrying *mcr-1* and *bla*_NDM-5_. Antimicrob Agents Chemother. 2017;61(12):e01757-17. 10.1128/AAC.01757-17 PMC5700326 28971871

[98] Bi Z, Berglund B, Sun Q, et al. Prevalence of the *mcr-1* colistin resistance gene in extended-spectrum beta-lactamase-producing *Escherichia coli* from human faecal samples collected in 2012 in rural villages in Shandong Province, China. Int J Antimicrob Agents. 2017;49(4):493–497. 28263896 10.1016/j.ijantimicag.2016.12.018

[99] Novem V, Shui G, Wang D, et al. Structural and biological diversity of lipopolysaccharides from *Burkholderia pseudomallei* and *Burkholderia thailandensis*. Clin Vaccine Immunol. 2009;16(10):1420–1428. 19692625 10.1128/CVI.00472-08 PMC2756838

[100] Xu Y, Wei W, Lei S, et al. An evolutionarily conserved mechanism for intrinsic and transferable polymyxin resistance. MBio. 2018;9(2):e02317-17. 29636432 10.1128/mBio.02317-17 PMC5893884

